# The effect of heat stress on sugar beet recombination

**DOI:** 10.1007/s00122-020-03683-0

**Published:** 2020-09-29

**Authors:** Mikel Arrieta, Glenda Willems, Jérôme DePessemier, Isabelle Colas, Alexandra Burkholz, Aude Darracq, Sigrid Vanstraelen, Pieter Pacolet, Camille Barré, Paul Kempeneers, Robbie Waugh, Steve Barnes, Luke Ramsay

**Affiliations:** 1 Cell and Molecular Sciences, The James Hutton Institute, Invergowrie, Dundee, DD2 5DA UK; 2 Division of Plant Sciences, University of Dundee at The James Hutton Institute, Invergowrie, Dundee, DD2 5DA UK; 3 SESVanderHave, Soldatenplein 15, 3300 Tienen, Belgium

## Abstract

Meiotic recombination plays a crucial role in plant breeding through the creation of new allelic combinations. Therefore, lack of recombination in some genomic regions constitutes a constraint for breeding programmes. In sugar beet, one of the major crops in Europe, recombination occurs mainly in the distal portions of the chromosomes, and so the development of simple approaches to change this pattern is of considerable interest for future breeding and genetics. In the present study, the effect of heat stress on recombination in sugar beet was studied by treating *F*_1_ plants at 28 °C/25 °C (day/night) and genotyping the progeny. *F*_1_ plants were reciprocally backcrossed allowing the study of male and female meiosis separately. Genotypic data indicated an overall increase in crossover frequency of approximately one extra crossover per meiosis, with an associated increase in pericentromeric recombination under heat treatment. Our data indicate that the changes were mainly induced by alterations in female meiosis only, showing that heterochiasmy in sugar beet is reduced under heat stress. Overall, despite the associated decrease in fertility, these data support the potential use of heat stress to foster recombination in sugar beet breeding programmes.

**Electronic supplementary material:**

The online version of this article (10.1007/s00122-020-03683-0) contains supplementary material, which is available to authorized users.

## Introduction

Sugar beet (*Beta vulgaris* ssp*. vulgaris*) is one of the major crops in Europe, providing nearly 20% of the world´s sugar production (Statistical Office of the European Communities 2018). It is a source of animal feed and is a feedstock for 30% of the bioethanol produced in Europe (Salazar-Ordóñez et al. 2013). The species has an estimated genome size of around 750 Mb (Arumuganathan and Earle 1991; Dohm et al. 2014), with a diploid complement of 2*n* = 18 chromosomes.

The original sugar beet breeding pool has been considered genetically narrow given its single origin in white fodder beet (Frese et al. 2009), and there has been a concerted effort in recent decades to broaden the crop’s gene pool. While the introgression of traits from wild relatives, in particular for biotic stress resistance, has been successful (Monteiro et al. 2018), it has also reportedly resulted in the introduction of undesirable traits from the exotic germplasm though linkage drag (Panella and Lewellen 2007).

Recombination, associated with crossing over (CO) during meiosis, fulfils an essential role in plant breeding by reshuffling portions of homologous chromosomes and creating new combinations of alleles. The lack of CO in genomic regions restricts breeding potential as loci remain tightly linked (Wijnker and de Jong 2008). Thus, a better control of this process, by either increasing the number of crossovers or modifying their distribution, could improve breeding programmes by, for example, enhancing breeders’ ability to remove deleterious alleles. The mitotic karyotype of sugar beet and related species has been studied previously and consists of seven chromosomes exhibiting median centromeres, with the remaining two having sub-median centromeres (Bosemark and Bormotov 1971; Paesold et al. 2012; Levan 1942). Classical meiotic studies show a prevalence of rod bivalents with occasional ring bivalents during metaphase 1 (Rasmusson and Levan 1939; Levan 1942). Tsuchiya and Nakamura (1979) described distal crossovers in meiosis, showing the bivalents were only held together by the euchromatic distal arms during diakinesis and the presence of rod bivalents at metaphase 1. Such studies imply that in sugar beet there is approximately 1 CO per bivalent, with an occasional extra CO, giving a total of around 9–11 COs per meiotic cell.

As in other species (Colas et al. 2016) there are some inconsistencies between the cytological CO counts and the genetic map lengths published in sugar beet. Early generation molecular genetic maps (McGrath et al. 2007; Schneider et al. 2007) showed some concurrence with the cytology, with genetic maps of around 500–600 cM, that would match the expectation of 450–550 cM reasonably well, given a correspondence of 1 cytological CO to 50 cM. Mapping in a wide cross (sugar beet × table beet) with multiple marker types gave a genetic map length of 526.3 cM across the nine linkage groups (McGrath et al. 2007), and SNP-based maps created from three different F_2_ populations gave maps of 507.1, 599.8 and 636.6 cM, with an integrated map of 664.3 cM (Schneider et al. 2007). However, the addition of larger sets of markers, despite forming genomic landmarks for sequencing, led to the inflation of the so-called K1 genetic map of Schneider et al. (2007) from 599.8 to 886.9 cM (Dohm et al. 2012) with most additional loci being interstitial rather than terminal compared to earlier SNPs (Schneider et al. 2007). More recent publications have inflated this further to 1141.4 cM (Holtgräwe et al. 2014), anchoring many novel genetic markers but, potentially, substantially over-estimating the recombination frequency in the species. Both the number and distribution of crossovers indicate that recombination overall is limited in sugar beet and that the reduced pericentromeric recombination may not correlate with a lack of gene content (Dohm et al. 2014). These features will limit the precision of introgression breeding and the mapping and cloning of genes located in these genomic areas.

A potential means of modifying crossover frequency and distribution of recombination is through the temporal application of different abiotic stressors. For example, plasticity of recombination under temperature stress has been reported in the literature, albeit with a varied effect in different species (Table 1). The positive correlation of recombination with temperature found in some species such as barley (Phillips et al. 2015; Higgins et al. 2012), wheat–rye hybrids (Kato and Yamagata 1982) and Arabidopsis (Modliszewski et al. 2018) contrasts with a negative correlation in others such as in wheat (Bayliss and Riley 1972) or wild garlic (Loidl 1989). However, the effects described in these studies are difficult to compare given the different experimental designs (Wilson 1959a, b) and methodologies (Table 1). Nevertheless, the potential of temperature effect to be exploited in breeding programmes by increasing the recombination in interstitial regions in species like barley (Phillips et al. 2015) provides the rationale for exploring whether similar effects could be obtained in pericentromeric recombination-cold genomic regions of sugar beet.

**Table 1:** Studies on the effect of temperature on recombination (frequency and distribution) in different species and the direction of the effect (increase/decrease) and change of distribution, if studied. The approach used is also indicated as “Cyt.” for cytological approaches, “Map.” if recombination was assessed by genetic mapping, and “Rep. lines”, if pollen reporter lines were used

Species	Inbred/outbred	Temp. stress	Temp. effect on CO freq	Temp. effect on CO distr	Meio. sex	Approach	References
*Allium ursinum*	Outbred	Heat	Decrease	–	Male	Cyt	Loidl (1989)
*Arabidopsis thaliana*	Inbred	Heat	Increase	–	Male	Rep. lines	Francis et al. (2007)
*Arabidopsis thaliana*	Inbred	Heat	Increase	–	Male	Cyt	Modliszewski et al. (2018)
*Arabidopsis thaliana*	Inbred	Heat	Increase	–	Male	Rep. lines	Lloyd et al. (2018)
*Arabidopsis thaliana*	Inbred	Cold	Increase	–	Male	Rep. lines	Lloyd et al. (2018)
*Endymion nonscriptus*	Outbred	Heat	Decrease	–	Male	Cyt	Wilson (1959b)
*Endymion nonscriptus*	Outbred	Cold	Increase	–	Male	Cyt	Wilson (1959b)
*Endymion nonscriptus*	Outbred	Heat	Decrease	–	Male	Cyt	Wilson (1959a)
*Endymion nonscriptus*	Outbred	Heat	Decrease	–	Male	Cyt	Elliott (1955)
*Fritillaria meleagris*	Outbred	Heat	Decrease	↑ Proximal	Male	Cyt	Barber (1942)
*Hordeum vulgare*	Inbred	Heat	Decrease	↑ Interstitial	Male	Cyt	Higgins et al. (2012)
*Hordeum vulgare*	Inbred	Heat	Increase	↑ Interstitial	Male	Map. and Cyt	Phillips et al. (2015)
*Hordeum vulgare*	Inbred	Heat	No Dif	No Diff	Female	Map	Phillips et al. (2015)
*Hyacinthus orientalis*	Outbred	Heat	Increase	–	Male	Cyt	Elliott (1955)
*Rhoeo spathacea* var. *variegata*	Outbred	Heat	Decrease	↓ Distal	Male	Cyt	Lin (1982)
*Tradescantia bracteata*	Outbred	Heat	Increase and then decrease	↑ Interstitial	Male	Cyt	Dowrick (1957)
*Triticum aestivum*	Inbred	Heat	Decrease	↑ Interstitial	Total	Map	Coulton et al. (2020)
*Triticum aestivum*	Inbred	Heat	Decrease	–	Male	Cyt	Bayliss and Riley (1972)
*Triticum aestivum nullisomic for chr.5D*	Inbred	Heat	Increase	–	Male	Cyt	Bayliss and Riley (1972)
*Triticum aestivum Ph1*	Inbred	Cold	Increase	–	Male	Cyt	Martín et al. (2017)
*Triticum aestivum* x *Secale cereale* F_1_s	Inbred	Heat	Increase	–	Male	Cyt	Kato and Yamagata (1982)
*Uvularia perfoliata*	Outbred	Heat	Increase and then decrease	↑ Interstitial	Male	Cyt	Dowrick (1957)
*Zea mays* T_5_-T_6c_ F_1_s	Outbred	Cold	Increase	–	Male	Cyt	Khan (1955)

**Fig. 1:**
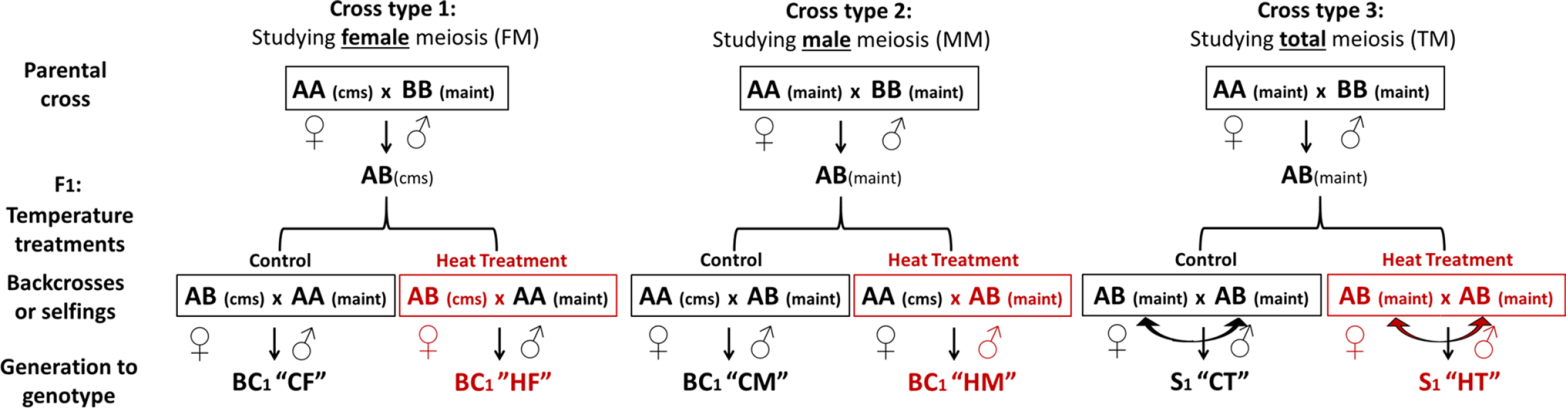
Diagrams summarizing the crosses involved in the temperature experiments to analyse female (FM), male (MM) and total meiosis (TM), respectively. The suffix in parenthesis indicates if the plant was male sterile (cms) or maintainer (maint), that is plant with a fertile cytoplasm but recessive alleles in the nucleus. The colour indicates where plants were grown, being black for control conditions and red for heat treatment. All the crosses produced six different populations; control female meiosis (CF), heat female meiosis (HF), control male meiosis (CM), heat male meiosis (HM), control total meiosis (CT) and heat total meiosis (HT)

The following work describes a study to address the possibility of using heat stress to manipulate recombination distribution using heterozygous F_1_ sugar beet plants that were stressed with high temperature conditions during meiosis, followed by performing reciprocal backcrosses (Fig. 1) to distinguish the effects on male and female meiosis. Reciprocal crosses took advantage of the cytoplasmic male sterility (CMS) widely used in commercial hybrid production (Panella and Lewellen 2007), which avoided the need to emasculate flowers under heat stress conditions. CMS is caused by the interaction of a male sterility-inducing mitochondria and at least two nuclear loci called restorer-of-fertility loci (Rf) (Arakawa et al. 2018; Moritani et al. 2013). A sugar beet line containing a sterile cytoplasm will only be sterile if the nuclear restorer is homozygous for the recessive alleles (rf rf). Normal cytoplasm plants with homozygous recessive Rf genes can be used as pollen donors to maintain CMS lines, when the cytoplasm is inherited from the female parent. This system is used to create maintainer lines that can be used to produce male sterile plants with the desired genotypes.

In this study, all the male parental lines used for creating the *F*_1_ plants for the experiment were double haploid (DH) maintainers of cytoplasmic male sterility. Finally, the recombination patterns were assessed by genotyping the Bc_1_ progeny and analysing the segregation of polymorphic SNP markers.

## Material and methods

### Plant material, temperature treatments and backcrosses

Crosses between double haploid lines (named AA and BB in Fig. 1) of sugar beet (*Beta vulgaris* ssp*. vulgaris;* 2*n* = 18) were used to generate different sets of heterozygous *F*_1_s. *F*_1_ plants were grown in control conditions at 20 °C until bolting (the start of the elongation of the primary axis) was observed (Mutasa-Göttgens et al. 2010) prior to any observable floral bud development and any meiosis. Individual plants were then moved to heat stress rooms at 28/25 °C day/night temperature with 16 h of photoperiod, with light supported by infrared lights, while other individual plants were kept in control conditions. The plants were kept in these conditions for six weeks to ensure the temperature treatments would completely cover meiosis starting from premeiotic stages. The backcrosses were made during the heat treatments, when floral buds were observed developing in the branches. Parental homozygous lines (AA) grown under control conditions were used as either male or female parent, and the same crossing regime was carried out under control conditions for the control populations. This way, *F*_1_ × AA was used to analyse female meiosis (FM) and AA × *F*_1_ to analyse male meiosis (MM). The *F*_1_ plants were allowed to self to produce *F*_2_ populations which were used for the analysis of total meiosis (TM). Plants were kept in their respective temperature treatments until seeds were developed.

### DNA extractions, genotyping and analysis

The crosses resulted in six different populations, three per treatment (control or heat): four Bc_1_ populations: control for female meiosis (CF), heat for female meiosis (HF), control for male meiosis (CM), heat for male meiosis (HM) and two *F*_2_ populations control for total meiosis (CT), and finally heat for total meiosis (HT). The amount of seed produced per plant from each population was weighed after harvesting and a subset selected for genotyping from two different individual plants per cross-type (three for HF due to limited seed availability). The differences in seed weight means per treatment and population were tested with a *t*-test and plotted using the ggpubr and ggplot packages in *R*.

Leaf tissue from glasshouse-grown BC_1_ and *F*_2_ plants was collected for DNA extraction using standard in-house protocols in SES Vanderhave, Tienen, Belgium. A total of 230 KASP (LGC) markers with known genomic positions that gave genome-wide coverage in elite material in-house were used to genotype the populations and the data generation, and allele calling was carried out using KRAKEN software (LGC Technologies). The physical positions of the markers, as well as the identification of the centromere flanking makers, were provided from in-house SES VanderHave data. The latter were obtained by the deduction of the centromere position, based on indirect estimators such as the conjunction of Gypsy element density maps, high-density marker genetic maps and gene density maps. After clustering, the data were imported into Excel, where the quality of the segregation patterns was checked and individual markers removed based on quality and monomorphism for the crosses and polymorphism heterogeneity between individuals. Only markers that worked well and showed polymorphism within the six populations (92 loci) were kept for the analysis and comparison of all the Bc_1_ populations, while more marker data (117 loci) were kept for the TM population comparison. This difference in marker numbers was due to a lack of polymorphism in a portion of the Bc_1_ populations suggesting that the individual plants that provided the parental lines used to generate the *F*_1_s were not totally homozygous/homogeneous.

MapDisto 2.0 (Lorieux 2012; Heffelfinger et al. 2017) was used to calculate the genetic maps, using the Kosambi mapping function with a minimum LOD score of 3 and a maximum recombination fraction of 0.37. Although the default marker order was taken into account, the AutoOrder function was used to reorder the markers within linkage groups. Using the calculated genetic distances, the genetic maps were represented and drawn together using Mapchart (Voorrips 2002). The differences between the genetic chromosomes and centromere flanking genetic intervals for each treatment were tested and plotted using the Wilcoxon’s signed rank test (as in Devaux et al. 1995) with the R ggpaired function and ggpubr and ggplot packages.

The significance of the temperature treatment and sex on recombination was tested using Genstat (VSNi) by fitting generalized linear models with a Poisson distribution with sex, heat treatment and chromosome (and their interactions) as fixed effects and marker interval as a random effect. The differences between the chromosomes and centromere flanking genetic intervals for each treatment were also tested and plotted using the Wilcoxon's signed rank test (as in Devaux et al. 1995) with the R ggpaired function and ggpubr and ggplot packages. Differences in recombination between the pericentromeric region for each of the chromosomes under the different treatments were also assessed by comparing the number of crossovers per individual for each treatment by a *χ* squared test with a contingency table.

### Cytology

Inflorescence branch material measuring approximately 15 cm was collected and fixed in 3:1 ethanol/acetic acid (EtOH/AA) fixative in 50-mL Greiner centrifuge tubes (Sigma-Aldrich) and stored at 4 °C. After two weeks, the fixative was replaced with 70% ethanol for longer-term storage. Anthers were dissected using a stereo microscope (LEICA) and staged with a light microscope (Olympus CK2) using aceto-carmine staining. Metaphase spread slides were prepared as described by Higgins et al. (2012) with modifications: Counterstaining solution with HOECHST 33,342 and mounting in Vectashield (H-1000) was sealed with nail varnish as described in Colas et al. (2016). The differences between the counts in each treatment were compared using the R ggpubr and ggplot packages.

## Results

### Cytology of male meiosis in sugar beet

**Fig. 2:**
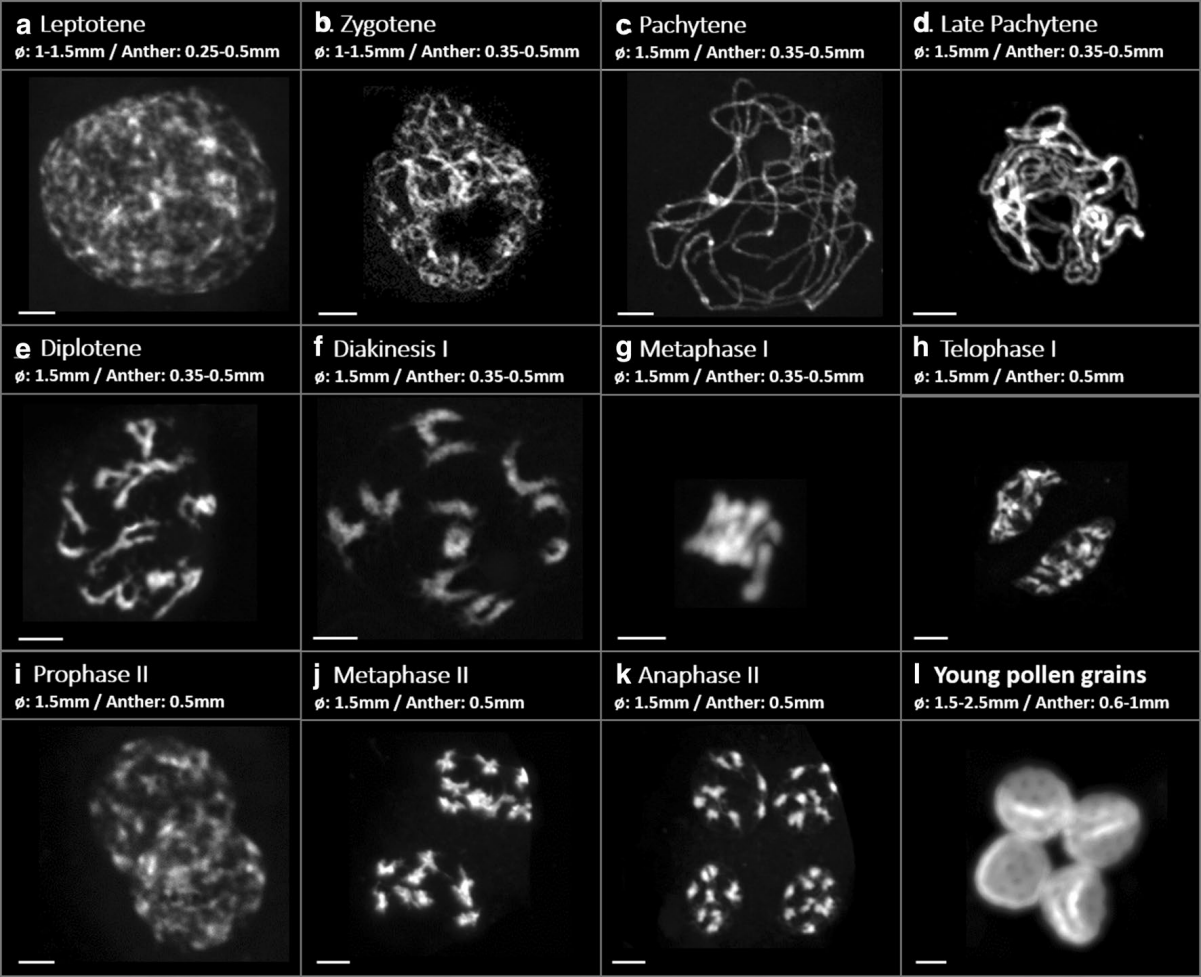
Meiotic stages found in different anther sizes (mm) and flower diameter (mm). Rod bivalents can be observed during diakinesis and metaphase 1. Bar size: 5 µm

Fixing inflorescence branches provided material to allow the progression of meiosis under control conditions to be followed (Fig. 2). Visualization was achieved following anther dissection and squashing of different sized flower buds. An overall correlation was found between flower bud diameter, dissected anther size and the meiotic stages observed (Fig. 2). The first meiotic division (Fig. 2a–h) and the second (Fig. 2i–k) were observed in flower buds of 1.5 mm with earlier stages found in anthers sizes of 0.25–0.5 mm.

**Fig. 3:**
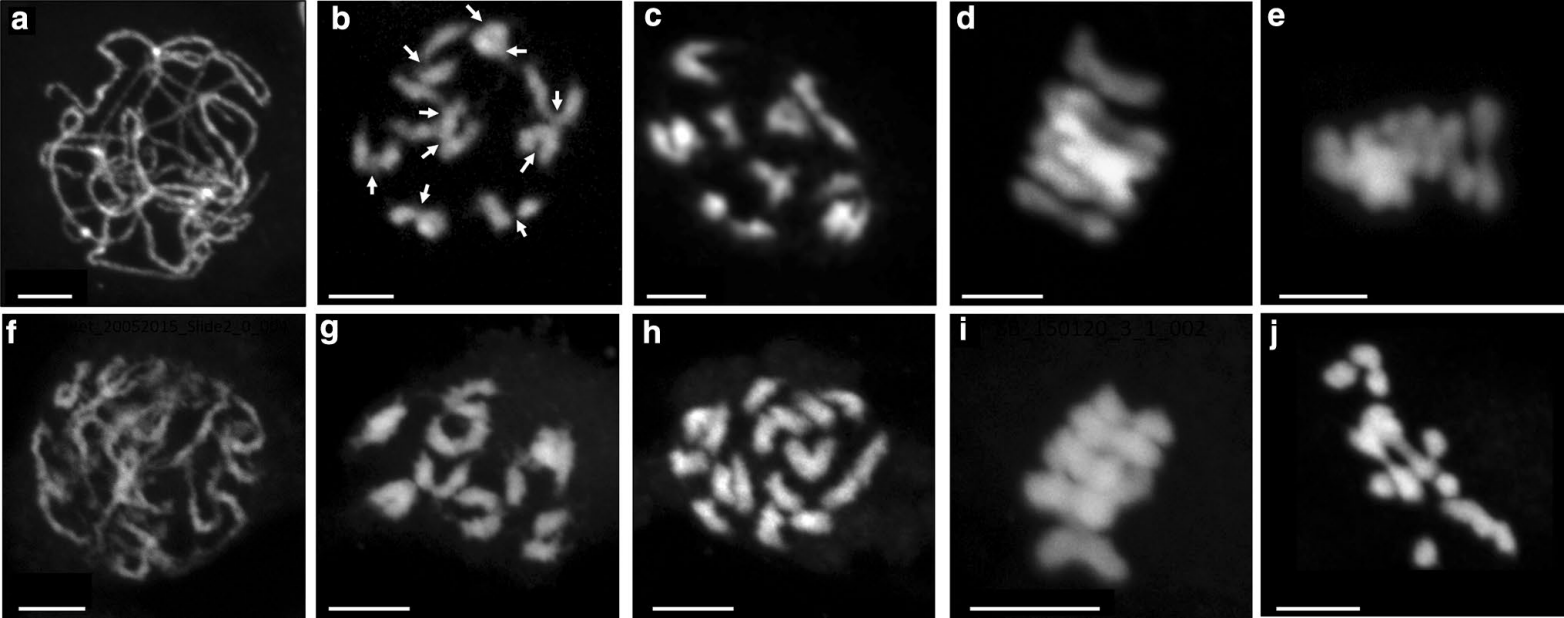
Pictures of pachytene (**a**,** f**), diakinesis (**b**,** c**, **g**,** h**) and metaphase 1 (**d**,** e** ,**i**,** j**) in control (**a**–**e**) and heat treatment (**f**–**j**) conditions. Arrows in **b** indicate crossovers (CO counted at diakinesis stage). Bar size: 5 µm

We observed classical changes in chromosome structure expected with nine pairs of chromosomes visible at metaphase II (Fig. 2j). Nine bivalents were also visible at metaphase I (Figs. 2g, 3d,e) but clearer at diakinesis where the chromosomes were less clustered (Figs. 2f, 3b–c). The bivalent structure apparent at diakinesis indicated that most were rod bivalents with the chromosomes held by a single CO in only one arm rather than ring bivalents held by CO in both arms (Fig. 3b). Cytological preparations of anthers from heat-stressed inflorescences indicated that critical stages of meiosis I were similar in control (Fig. 3a–e) and heat-stressed conditions (Fig. 3f–j).

The spread of bivalents at diakinesis allowed an estimation of the number of chiasmata (the physical manifestation of CO) per cell for male meiosis in both control and heat-stressed plants.

**Fig. 4:**
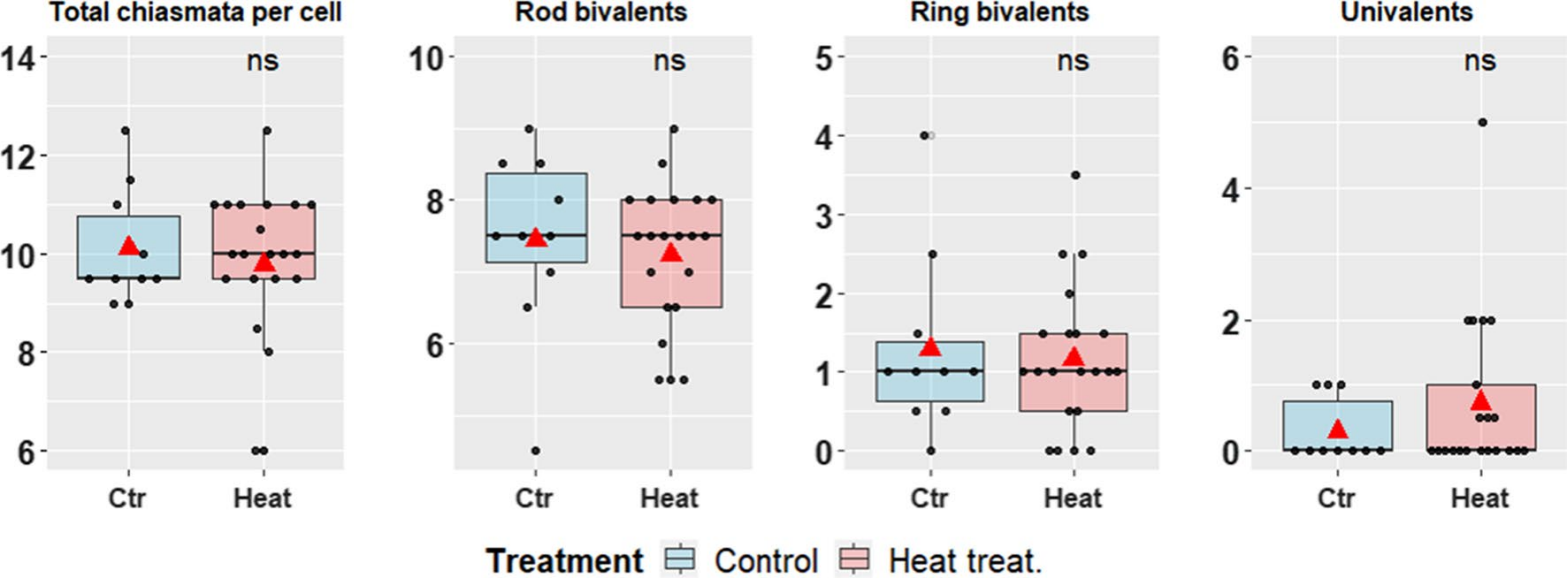
Boxplots from left to right: Male cytology chiasmata counts per cell (**a**), the number of rod (**b**), ring bivalents (**c**) and univalents (**d**) found for each treatment. Significance of t-test shown as: “ns” for non-significant. The red triangle represents the mean of the samples

The analysed images of 10 and 21 cells for control and heat treatment, respectively, showed no significant difference in the total chiasmata numbers between treatments, with an average of 10.1 and 9.8 chiasmata for control and heat treatment, respectively (Fig. 4a). There was a predominance of rod bivalents for both treatments (74% for both control and heat treatment) followed by ring bivalents (13% and 12%), finally 3% and 8% of univalents (no CO) (Fig. 4b–d); however, none of these differences between treatments were found to be statistically significant. The presence of univalents was unexpected, especially in control plants, and could possibly represent artefacts due to breaking of the chiasmata of distal rod bivalents during spreading.

### Population development

During the vegetative growth of the plants in control conditions prior to the induction of the heat stress, a yellowing of leaves of the plants was observed presumably due to a virus infection. This may have reduced fertility but would have affected all the derived populations equally. A significant reduction in seed weight was found between the populations due to the effect of the applied heat stress and also due to the differential between backcross and selfed-derived populations (Supplementary Figure S1, Table S1). The fewest seeds were obtained from the backcrossed populations under heat stress, with the plants used to assess female meiosis (HF) having the lowest number of seeds (165 seeds) followed by the male meiosis (HM) plants (373 seeds) (Supplementary Figure S2).

### Heat stress induces higher total and pericentromeric recombination

Recombination frequency and distribution on TM populations (total meiosis) was studied by genotyping using 117 polymorphic markers, while for the comparison between the different meioses (MM and FM), the 92 markers that were informative for all the three populations were considered, dropping 25 markers from previous analysis. As described above, populations of different sizes for CF, HF, CM, HM, CT, HT, comprising 354, 156, 437, 213, 520 and 520 individualsrespectively, were used for genetic analysis. Marker representation on each of the chromosomes was not even, with some chromosomes being better represented than others. All chromosomes had at least 7 markers and covered at least 90% of the physical length of the chromosomes, except for chromosome 9 where 80% of the chromosome was represented, and chromosome 7, which had only 3 informative markers covering only 2% of the physical distal arm. Chromosome 4 had a large interval without markers (between the positions 6% and 93% of the physical chromosome) and separated into two different linkage groups during genetic analysis of HM and HF; these were joined with a distance of 50 cM for the calculations. The reordering of the markers was not entirely consistent with the reference order having some subtle changes between markers that were very close to each other physically, but these did not change substantially the length of the maps. Chromosome 9 was the most consistently affected by reordering. This chromosome also had a large genomic region unexpectedly lacking recombination, suggestive of the presence of a rearrangement in the short arm of the chromosome between the two parental lines AA and BB (Supplementary Figure S3). Segregation distortion was found in the different populations and between treatments (Supplementary Table S2) with chromosome 5 in particular having distorted markers for all populations, specially under heat treatment. In general, the backcrosses showed more regions with distortion than the selfed F_1_s. Chromosome 9 showed a significant distortion in CM but interestingly not in HM.

The significance of the treatment and chromosome on recombination was tested with a generalized linear mixed model (GLMM) across all the marker intervals for total meiosis data (CT and HT) and indicated a significant effect of temperature (*p* < 0.01) (Supplementary Table S3). Chromosome 7 was excluded from the analysis given that the low number of markers was not informative. A similar analysis was undertaken regarding female and male meiosis (CF, HF, CM and HM) including sex as another fixed effect. This test showed a significant effect of the sex (*p* < 0.05) and the interaction between sex and temperature (*p* < 0.05) (Supplementary Table S4) among all chromosomes together and among the intervals within each chromosome. Additional GLMMs were tested, but no significant effect was found for each chromosome independently.

**Fig. 5:**
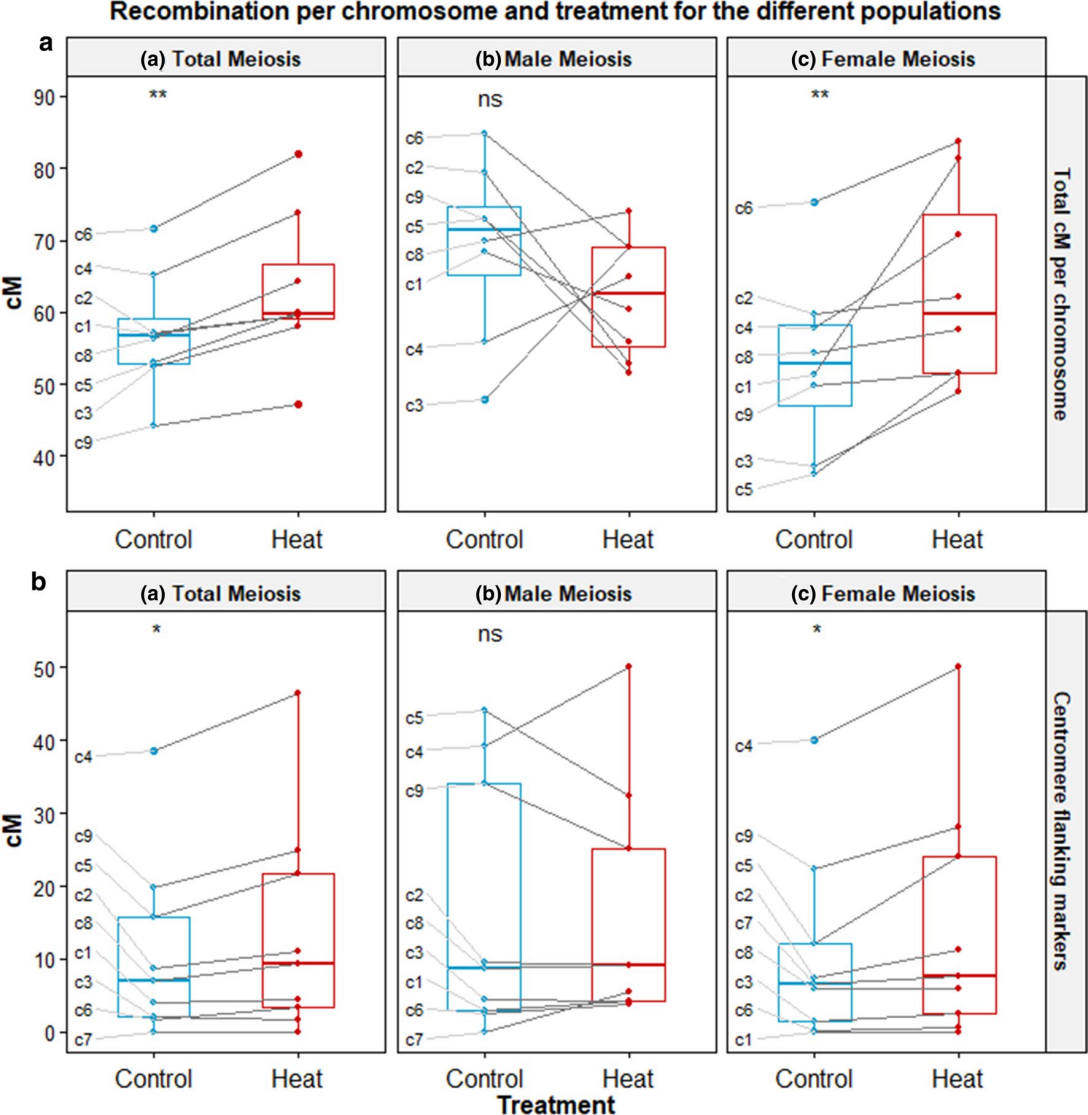
Genetic distances in cM for each chromosome for all the markers (**a**), and between the centromere flanking markers (**b**) under control (blue) and heat treatment (red), and for each population (**a**–**c**). The p value shown above each graph indicates the result of the comparison with the Wilcoxon signed rank test, being *P < 0.05 and **P < 0.01, and ns “not significant”. Chromosome 7 is not shown on section **a** graphs given its low coverage (3 markers) and values

**Fig. 6:**
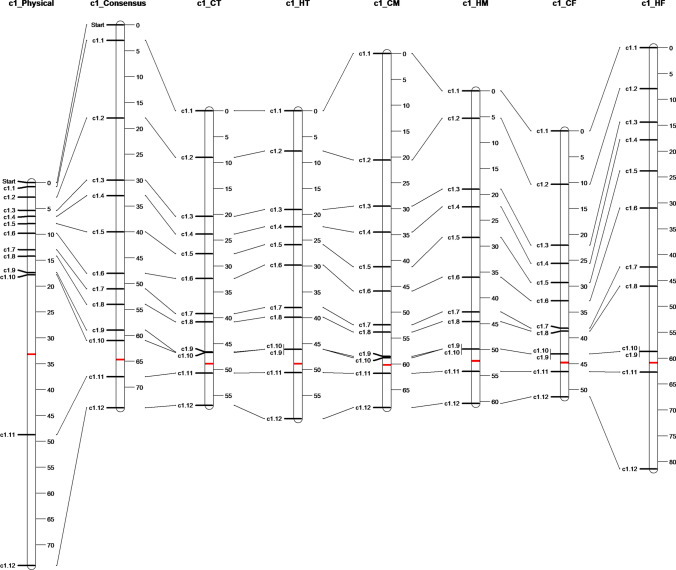
Comparison for chromosome 1 of the physical and consensus genetic maps with the genetic maps calculated for the different populations: Total meiosis control (CT), total meiosis heat shock (HT), control male meiosis (CM), heat-treated male meiosis (HM), control female meiosis (CF) and heat-treated female meiosis (HF). The physical chromosome map shows the proportional position of the markers when physical chromosome is scaled to the same size as the corresponding consensus map (units not in Mbp). The approximate position of the centromere is marked with a red bar

The effect of temperature on the genetic length of the chromosomes was also tested by the Wilcoxon signed rank test finding significant increases in the plants derived from heat treatment compared to control for total meiosis (511.3:463.7 cM, *p* < 0.01) and in female meiosis (516.0:431.1 cM, *p* < 0.01), but not for male meiosis (503.3:559.2 cM *p* > 0.05) (Figs. 5a,  6, and the supplementary Table S5). The reduction of recombination in the male meiosis under heat treatment was not consistent for all the chromosomes, as chromosomes 3, 4 and 8 showed the opposite pattern (Table 2, Supplementary Figure S4). The increase with heat for female meiosis and total meiosis was consistent for all chromosomes, with the exception of the markers on chromosome 7 in total meiosis. These differences would be translated into an average 1.7 CO more per meiotic cell under heat-treated conditions for female meiosis, while male meiosis would show a reduction of 1.1 COs and total meiosis an increase of 0.95 CO. All genetic distances between markers are summarized in the supplementary data Table S6.

**Table 2:** Comparison of the genetic distances (cM) for the total maps and between the markers that were flanking the centromeres. The number of loci in brackets refers to the male and female populations

Total map								Centromere flanking markers						
Individuals		Total meiosis		Male meiosis		Female meiosis			Total meiosis		Male meiosis		Female meiosis	
		520	520	437	213	354	156		520	520	437	213	354	156
Chr	Loci	Control	Heat treat	Control	Heat treat	Control	Heat treat	Loci	Control	Heat treat	Control	Heat treat	Control	Heat treat
*Genetic distance (cM)*														
c1	12	56.9	59.5	68.4	60.4	51.4	81.4	2	4	4.5	3	4.3	0	0
c2	11	57.1	59.6	79.5	52.8	59.7	62.2	2	8.6	11.1	9.6	9.2	7.4	11.4
c3	17 (8)	52.3	58	47.9	69.1	38.6	49	2	2.2	1.8	4.4	4.3	1.4	2.6
c4	11 (7)	65.2	73.9	55.8	64.9	57.8	70.8	2	38.5	46.4	39.2	50	40.1	50
c5	10 (7)	53	60.1	72.9	55.9	37.5	51.5	2	15.7	21.8	44	32.4	12.2	24
c6	20 (14)	71.6	82.1	84.9	69.1	75.3	83.7	3	1.8	3.3	2.6	3.8	0.3	0.6
c7	3	7.1	6.5	6.8	5.5	6.7	8.4	2	0	0	0	5.5	6.7	7.8
c8	18 (15)	56.3	64.3	70	74.1	54.3	57.5	5	7	9.4	8.7	9.2	5.8	6
c9	15	44.2	47.3	73	51.5	49.8	51.5	3	19.8	24.9	34	25	22.4	28.2
Total	117 (92)	463.7	511.3	559.2	503.3	431.1	516	23	97.6	123.2	145.5	143.7	96.3	130.6

In order to assess whether heat increased recombination in the recombination poor regions, we focused on assessing recombination between markers available flanking the centromeres of each chromosome. Notwithstanding that the intervals are different in each chromosome depending on the markers available, a significant pattern of higher recombination rate in the heat-derived population was observed for total (*p* < 0.05) and female meiosis (*p* < 0.05) by the Wilcoxon signed rank test, as indicated in Fig. 5. The sum of male and female meiosis does not quite equal the total meiosis, presumably due to differences in the F_1_ individuals sampled for the different populations.

**Fig. 7:**
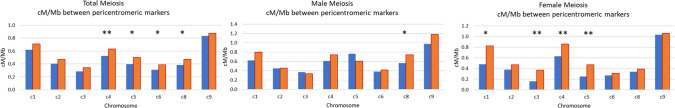
cM/Mbp between the centromere-flanking-markers (taking intervals of around 30 cM from consensus map) under control conditions (blue) and heat treatment (red). The proportion of crossovers/individuals was compared between treatments for each chromosome with a *χ* squared test (* < 0.05, ** < 0.01). Chromosome 7 was excluded given it had no markers flanking the centromere

The influence of temperature on pericentromeric recombination rates for each chromosome separately was independently compared with a *χ* squared test using intervals of approximately 30 cM from the consensus map that spanned the pericentromeric region where possible. This analysis showed an overall increase in recombination with temperature treatment for all chromosomes in all populations except chromosome 5 in male meiosis. These increases were significant for chromosomes 4, 5, 6 and 8 in total meiosis; and 1, 3, 4 and 5 for female meiosis, while only for chromosome 8 in male meiosis (Table 3 and Fig. 7).

**Table 3:** Summary of the crossovers (COs) per number of individuals (n) between pericentromeric markers for all chromosomes except 7 (which had no markers flanking the centromere) for all populations. The size of the iterval studied in each chromosome is indicated in Mbp, cM, and as percentage of the physical crhomosome. The comparison between treatments per chromosome and population was made by a χ squared test with contingency table (* < 0.05, ** < 0.01)

Chr	Mbp	% of the chr	cM of Consensus map	Total meiosis						Male meiosis						Female meiosis					
				Control		Heat treat				Control		Heat treat				Control		Heat treat			
				COs	*n*	COs	*n*	Sig	*P* value	COs	*n*	COs	*n*	Sig	*P* value	COs	*n*	COs	*n*	Sig	*P* value
c1	32.0	55%	28	190	503	207	487		0.1288	84	427	53	211		0.1150	53	349	39	151	**	0.0048
c2	43.1	78%	25	168	502	192	500		0.1036	80	418	39	201		0.9377	56	345	30	150		0.3092
c3	41.2	76%	33	119	517	140	511		0.1058	65	429	29	209		0.6696	22	336	23	153	**	0.0026
c4	55.6	91%	50	260	496	311	514	**	0.0095	135	412	81	207		0.1171	113	337	66	149	*	0.0233
c5	48.2	81%	28	173	473	192	429	*	0.0124	149	422	59	207		0.0882	41	343	33	148	**	0.0033
c6	51.1	78%	31	156	509	192	511	*	0.0197	82	429	43	205		0.5816	48	351	24	154		0.5721
c8	49.5	85%	30	186	516	223	513	*	0.0150	117	430	74	211	*	0.0409	43	259	20	106		0.6031
c9	41.6	92%	28	306	510	319	512		0.4498	166	431	94	206		0.0874	141	348	63	152		0.8457

## Discussion

Cytological observations of male meiosis in sugar beet confirmed the limited number of chiasmata evident during meiosis, with a high prevalence of rod bivalents in accordance with previous studies (Tsuchiya and Nakamura 1979), although some ring bivalents were found as described by Levan (1945). In addition, the bivalents at diakinesis often showed fine bridges between the homologues that could suggest very distal chiasmata (Figs. 2f,  3b). The meiotic spreads showed the presence of univalents in some cells which was unexpected, especially in control plants, and this may represent artefacts due to breaking of distal chiasmata of rod bivalents during spreading. Interestingly, no significant difference was found in the chiasmata counts between control and heat-treated plants for male meioses, though slight differences were observed with, for example, the chromatin in heat-treated plants looking somewhat more diffuse, or sticky (Fig. 3d, i). The average counts of 10.1 and 9.8 chiasmata per cell for the control and heat treatment, respectively (Fig. 4a), would give an expectation for ~ 1.1 CO per chromosome (albeit derived from male meiosis only) translating to 55.6 cM per chromosome and a total genetic map length of ~ 500 cM over the nine chromosomes. These chiasmata counts may be a slight underestimate given problems of resolution and potential breakage of very distal chiasmata in the spreading of the chromosomes.

Genetic mapping using the backcross populations (CM and HM) allowed a comparison between the cytological and genetic estimates of CO frequency. The pattern shown was similar with a slight non-significant decrease in recombination under the heat treatment (Fig. 5). However, the number of COs derived from the genetic maps of male meiosis (11.2 and 10.1 for control and heat treated, respectively) was slightly higher than the chiasmata counts observed cytologically (10.1 and 9.8). Indeed, the genetic map lengths observed (and the derived CO counts) will be underestimates given issues of marker genome coverage, in particular for chromosome 7. Nevertheless the CO estimates from the two methodologies are not that dissimilar, especially in comparison with the disagreement between cytological and genetic mapping approaches observed in other species such as barley where the number of COs differ from 14 chiasmata estimated from seven ring bivalents in metaphase spreads to 22 calculated from genetic mapping (Colas et al. 2016).

Our data provide strong evidence for a significant increase in overall recombination frequency induced by heat stress in sugar beet (Supplementary Table S3, Fig. 5). This is despite the non-significant difference in recombination in male meiosis with a significant difference between the sexes and an interaction with heat treatment (Supplementary Table S4). The increase observed would lead on average to the formation of 0.95 extra crossover per cell compared to control conditions (an increase of 10.3%) which appears to be due to a preferential effect in female gamete meiosis. The heat-treated plants showed a significant increase of 19.7% in the female genetic map and a non-significant reduction of 9.9% in the male map (Table 2). The patterns of recombination observed mean that heterochiasmy described in other plants like barley (Devaux et al. 1995) and Arabidopsis (Vizir and Korol 1990) can also be observed in sugar beet, with higher crossover frequencies in male meiosis than in female under control conditions. However, under heat stress, sugar beet does not exhibit heterochiasmy, as an increase in recombination in female meiosis compared to male (Supplementary Figure S5) brings the recombination rates to parity. This increase in recombination frequency is associated with a change in distribution of recombination with consistently higher recombination levels in interstitial and pericentromeric regions as shown by the intervals bounded by the centromere flanking markers (Figs. 5b, 7). The observation of a more plastic female meiosis under elevated temperature contrasts with that found in barley, where the increases of crossover frequency and pericentromeric recombination under heat stress were detected only for male meiosis, with female meiosis unaffected, or even showing shorter maps (Phillips et al. 2015).

Sugar beet is a wind-pollinated outbreeding species (Dark 1971), but the ratio of male-to-female recombination rate under control conditions was 559.0/431.2 = 1.30 which is high for an out-crossing species which generally show ratios < 1 with higher rates of recombination in female than male meiosis (Lenormand and Dutheil 2005). The ratio under heat stress is 503.3/516.0 = 0.98 which is more typical for an outbreeder. The reduction of the male/female recombination ratio with heat stress potentially accords with the decrease in male recombination frequency observed in other outbred plants like *Fritillaria meleagris* (Barber 1942) or *Allium ursinum* (Loidl, 1989) (Table 1). This contrasts with selfing species like barley (Phillips et al. 2015) or *Arabidopsis* (Modliszewski et al. 2018) where higher recombination is observed in male meiosis under heat stress.

Lenormand (2003) hypothesized that heterochiasmy could be explained by haploid selection with the expectation that the sex experiencing the more intense haploid selection would recombine less and this was largely borne out by a literature survey (Lenormand and Dutheil 2005). It is difficult to see how the results of the present study comply with this hypothesis, given that in an open pollinated species male meiosis would be expected to experience more selection and show less recombination. Moreover, how this hypothesis relates to changes in heterochiasmy with heat stress is unclear, though it is evident that selection pressure does change as shown by the changes in segregation distortion shown in both male and female genetic maps (Supplementary Table S2). Furthermore, there is not a simple correlation in the changes of segregation distortion and increased heat stress nor is it evident that selection would be lower on the female side under such circumstances.

The increased recombination observed in heat-treated plants is potentially of importance for breeding in sugar beet given the relatively low levels of recombination observed in the crop. Not only did total recombination frequency increase by 10.3% but there was a change in recombination distribution towards the pericentric region of the genome. The centromeric and pericentric regions exhibit suppressed recombination and though enriched in *Gypsy*-like retrotransposons do contain coding genes (Dohm et al. 2014). Increased recombination in these regions opens up the potential for reducing linkage drag associated with introgressed genes of interest and breaking up suboptimal linkage blocks to facilitate breeding progress.

The backcrossing approach was effective for studying the differences between male and female meiosis under heat stress but crossing plants under stress conditions was more complicated than initially envisaged due to flowering synchrony problems and partial sterility induced by heat. However, the selfed plants showed better fertility rates even in the heat stress conditions. This suggests that, for the potential application of heat to promote recombination as a breeding tool, it would be much easier to let plants self-pollinate, both in terms of the effort required and fertility observed while still benefitting from the increase in recombination frequency and change in distribution.

## Electronic supplementary material

Below is the link to the electronic supplementary material.

Supplementary file1 (XLSX 918 kb)

Supplementary file2 (PDF 786 kb)
