# Supplementary material for: The effect of heat stress on sugar beet recombination
Source: Theor Appl Genet. 2020 Sep 29;134(1):81–93. doi: 10.1007/s00122-020-03683-0 (PMC7813734; doi:10.1007/s00122-020-03683-0)
Supplement: Supplementary file 2 — Supplementary file2 (PDF 786 kb) [file 122_2020_3683_MOESM2_ESM.pdf]

Supplementary data

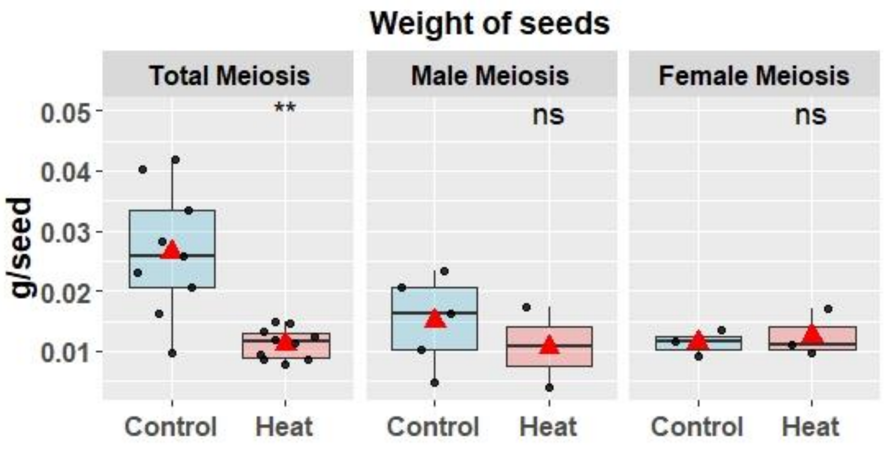

Figure S1: Mean weight of the harvested seeds per treatment and meiosis. Significance of t-test shown as: "ns" for non-significant, **P*<0.05, and **P*<0.01. The red triangle represents the mean of the samples.

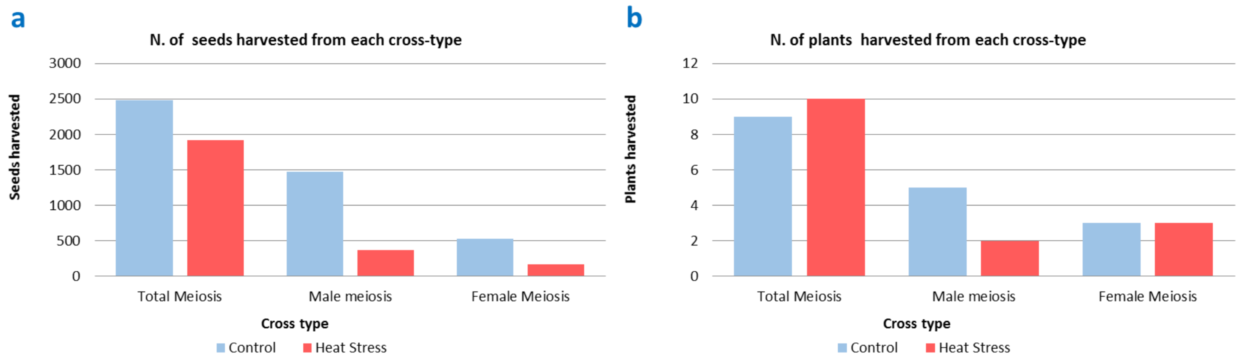

Figure S2: **(a)** the number of seeds harvested per cross-type and treatment, and (b) the number of plants harvested per cross and treatment.

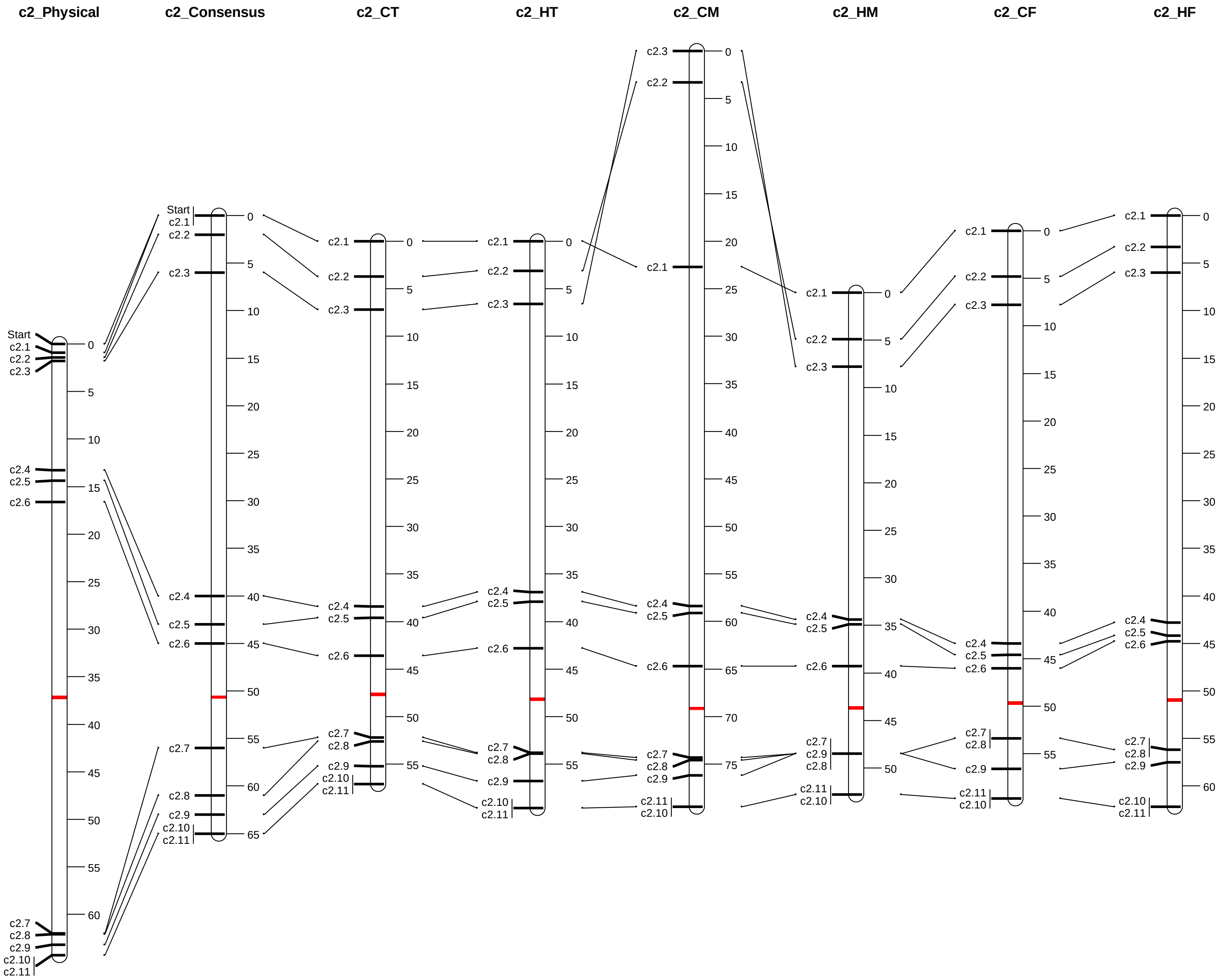
c2_Physical
c2_Consensus
c2_CT
c2_HT
c2_CM
c2_HM
c2_CF
c2_HF
Start
c2.1
c2.2
c2.3
c2.4
c2.5
c2.6
c2.7
c2.8
c2.9
c2.10
c2.11

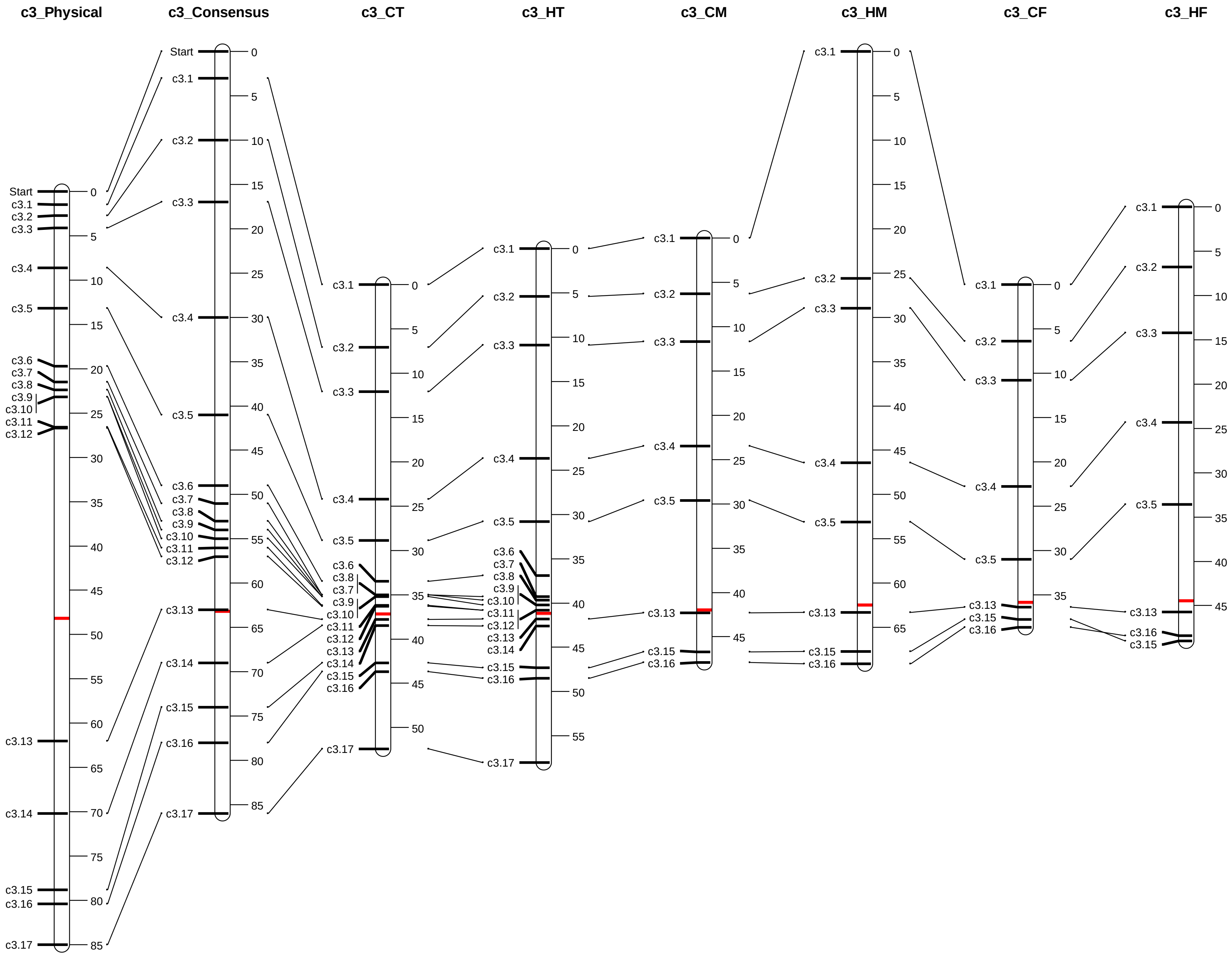
c3_Physical
c3_Consensus
c3_CT
c3_HT
c3_CM
c3_HM
c3_CF
c3_HF

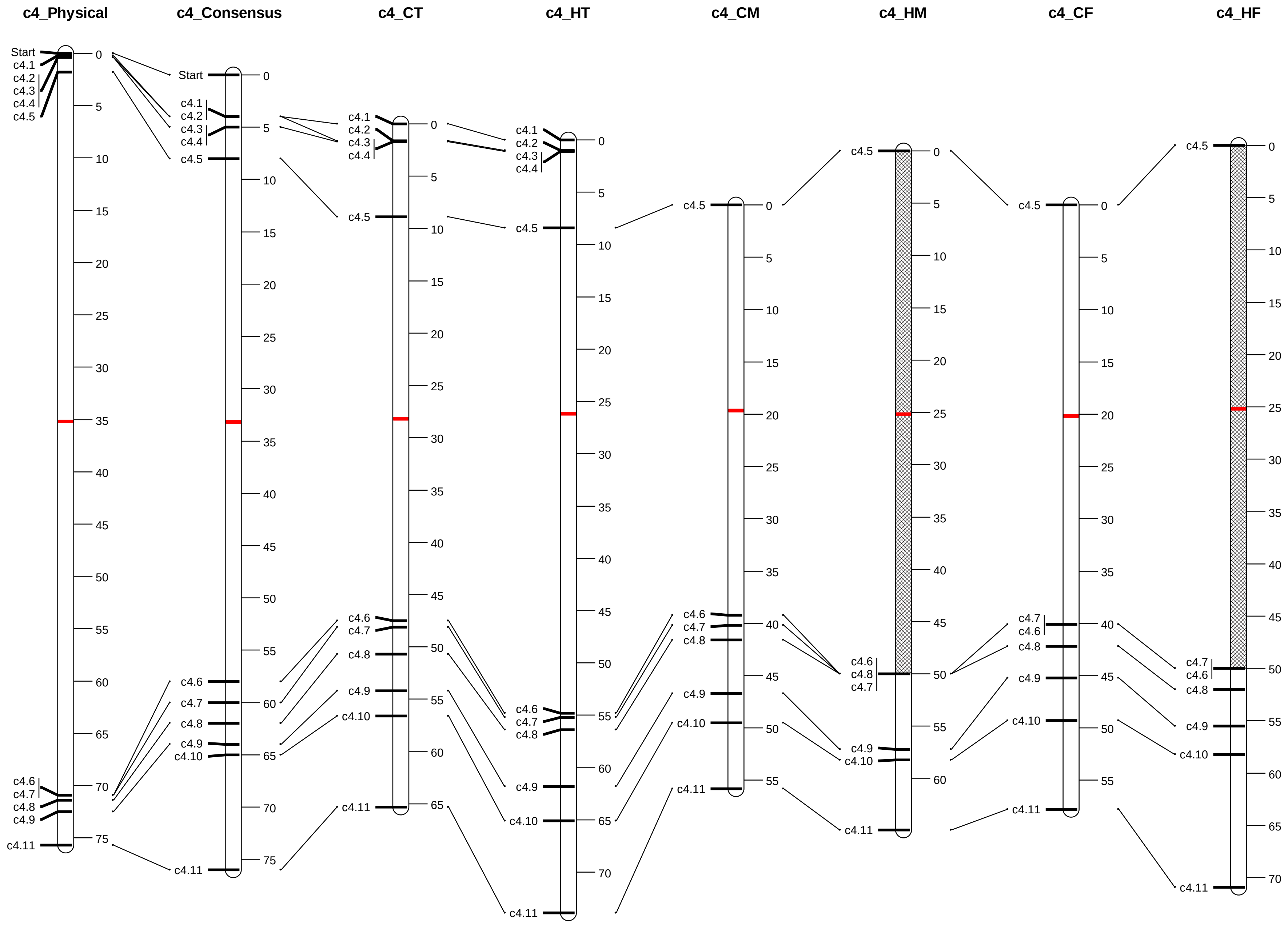
c4_Physical
Start
c4.1
c4.2
c4.3
c4.4
c4.5
c4.6
c4.7
c4.8
c4.9
c4.11
c4_Consensus
Start
c4.1
c4.2
c4.3
c4.4
c4.5
c4.6
c4.7
c4.8
c4.9
c4.10
c4.11
c4_CT
c4.1
c4.2
c4.3
c4.4
c4.5
c4.6
c4.7
c4.8
c4.9
c4.10
c4.11
c4_HT
c4.1
c4.2
c4.3
c4.4
c4.5
c4.6
c4.7
c4.8
c4.9
c4.10
c4.11
c4_CM
c4.5
c4.6
c4.7
c4.8
c4.9
c4.10
c4.11
c4_HM
c4.5
c4.6
c4.8
c4.7
c4.9
c4.10
c4.11
c4_CF
c4.5
c4.7
c4.6
c4.8
c4.9
c4.10
c4.11
c4_HF
c4.5
c4.7
c4.6
c4.8
c4.9
c4.10
c4.11

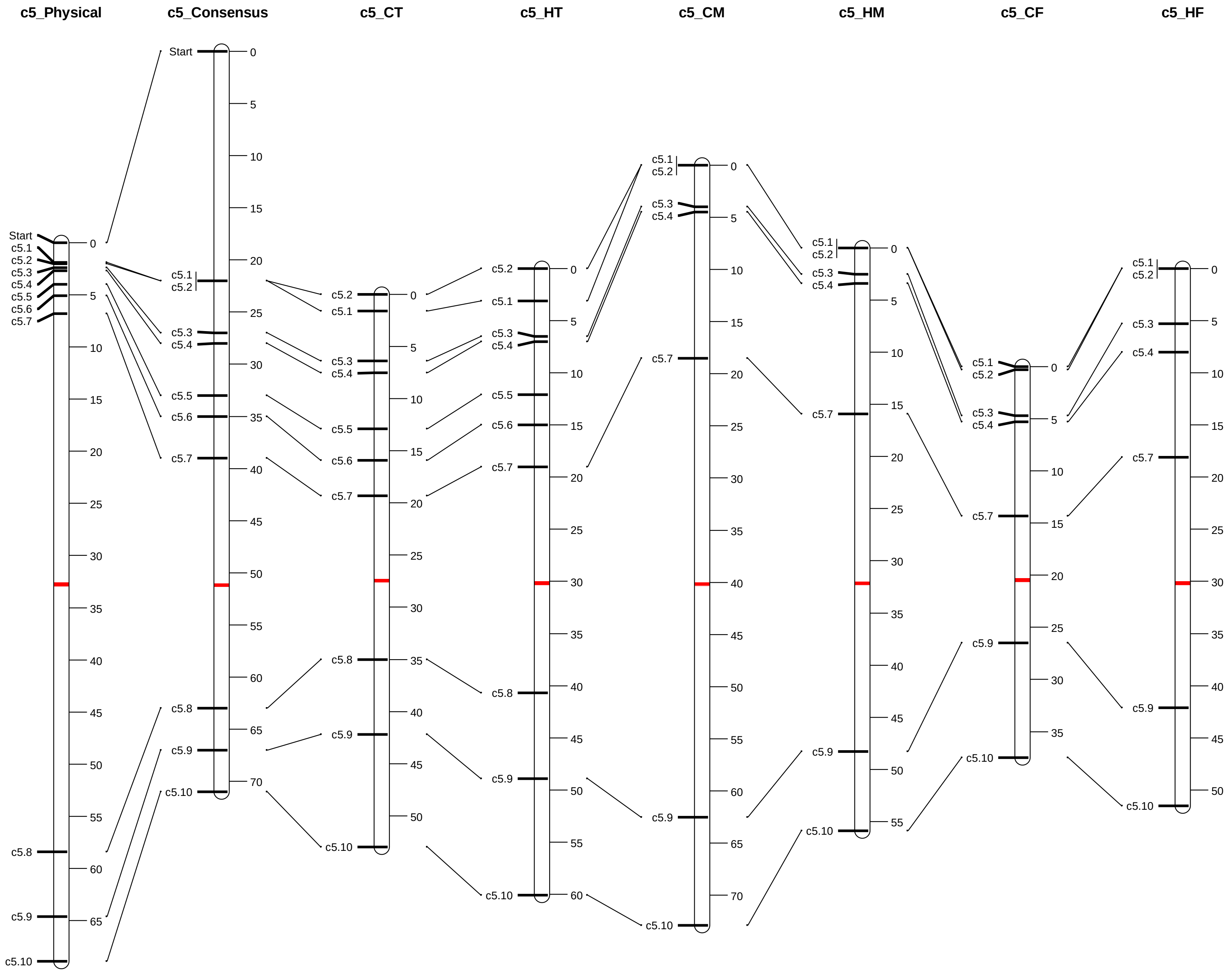
c5_Physical
c5_Consensus
c5_CT
c5_HT
c5_CM
c5_HM
c5_CF
c5_HF
Start
c5.1
c5.2
c5.3
c5.4
c5.5
c5.6
c5.7
c5.8
c5.9
c5.10

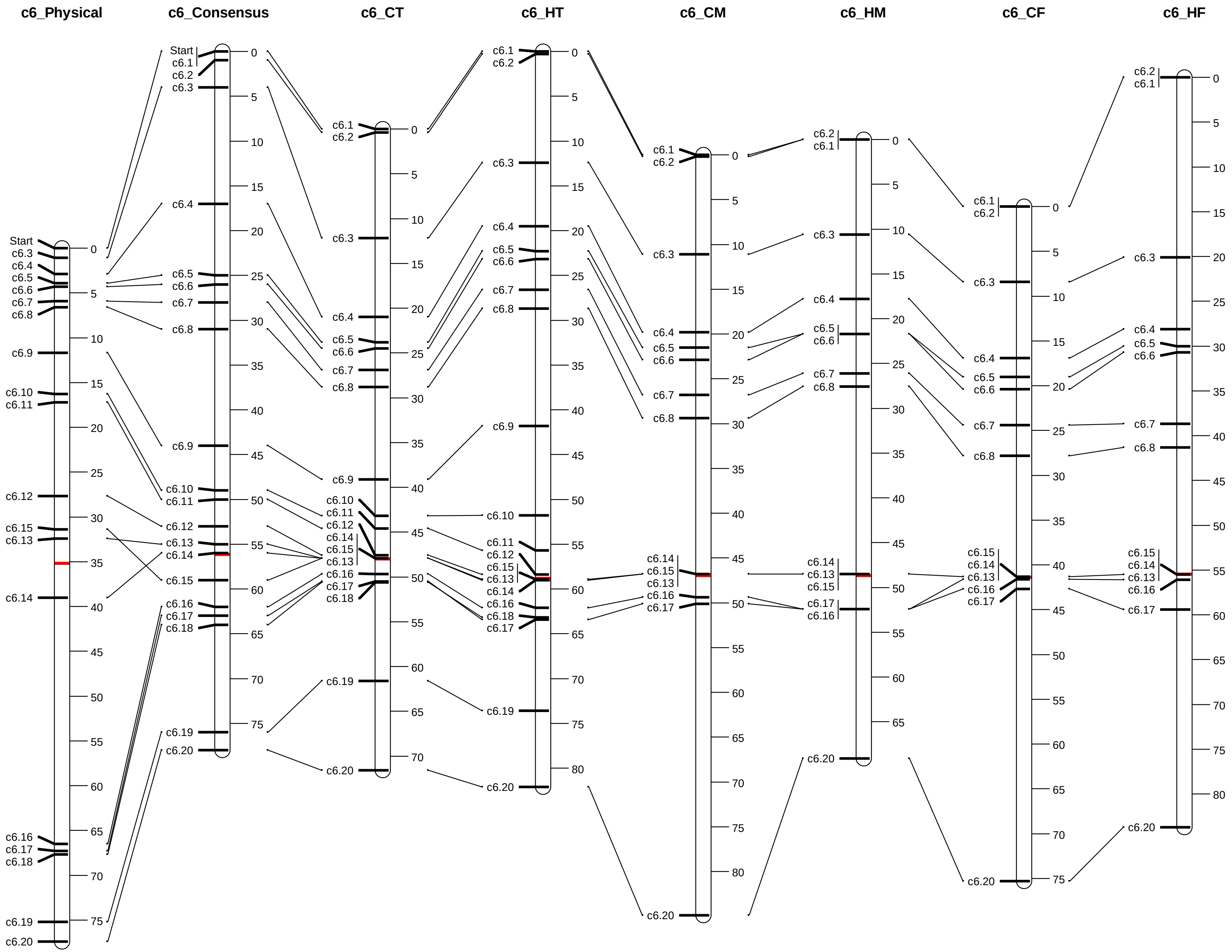
c6_Physical
c6_Consensus
c6_CT
c6_HT
c6_CM
c6_HM
c6_CF
c6_HF
Start
c6.1
c6.2
c6.3
c6.4
c6.5
c6.6
c6.7
c6.8
c6.9
c6.10
c6.11
c6.12
c6.13
c6.14
c6.15
c6.16
c6.17
c6.18
c6.19
c6.20

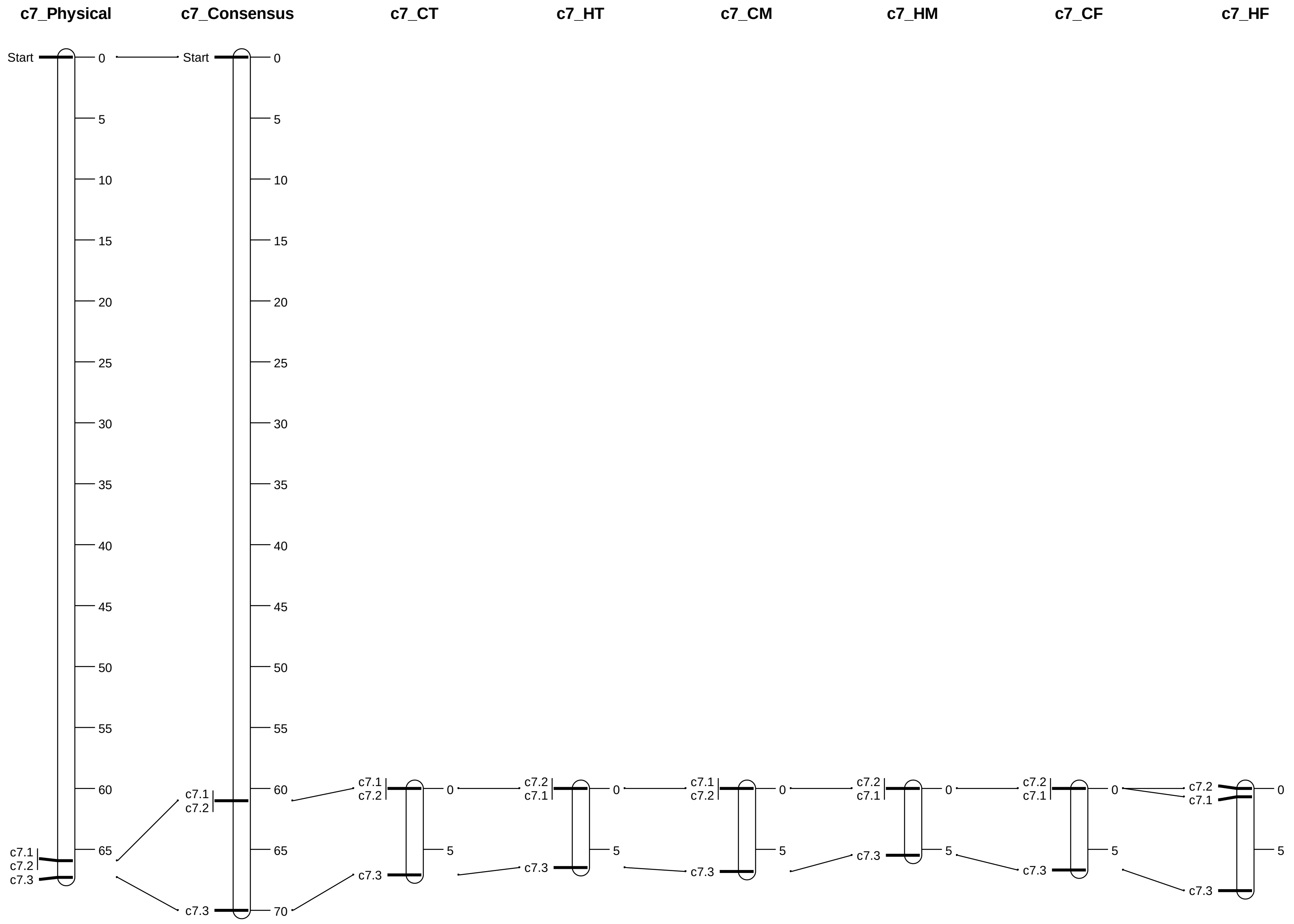

c7_Physical
c7_Consensus
c7_CT
c7_HT
c7_CM
c7_HM
c7_CF
c7_HF
Start
c7.1
c7.2
c7.3
0
5
10
15
20
25
30
35
40
45
50
55
60
65
70

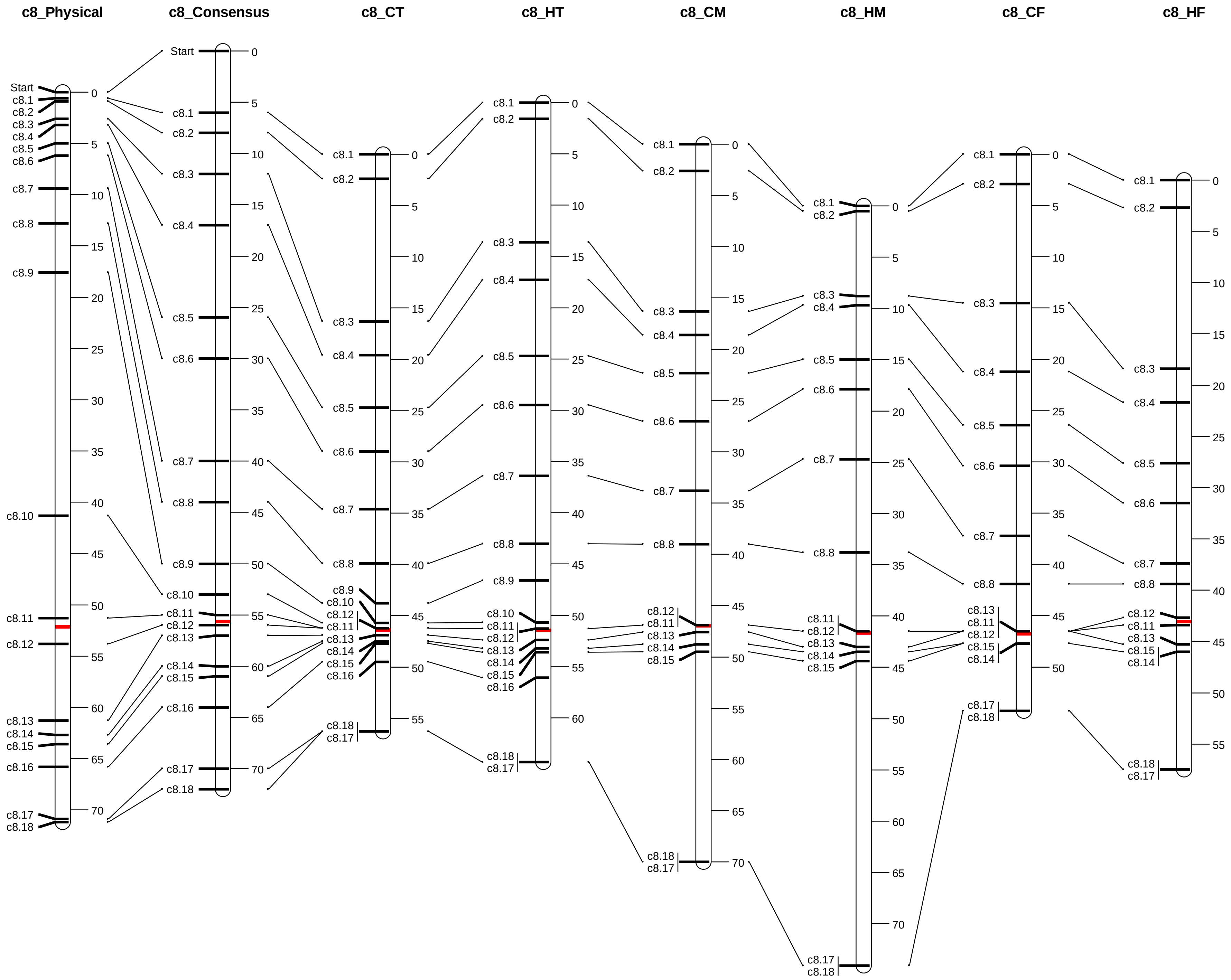
c8_Physical
c8_Consensus
c8_CT
c8_HT
c8_CM
c8_HM
c8_CF
c8_HF
Start
c8.1
c8.2
c8.3
c8.4
c8.5
c8.6
c8.7
c8.8
c8.9
c8.10
c8.11
c8.12
c8.13
c8.14
c8.15
c8.16
c8.17
c8.18

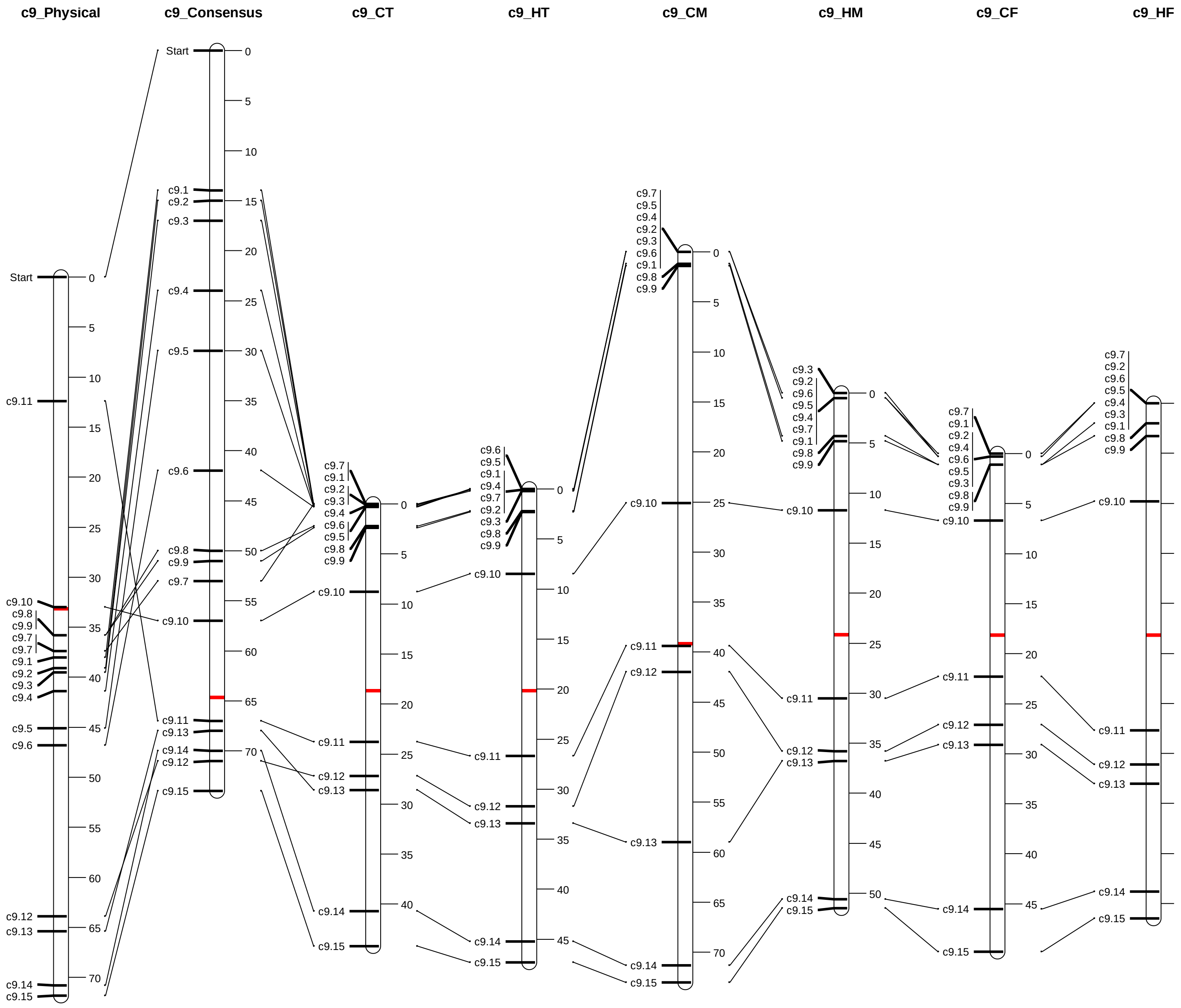
c9_Physical
c9_Consensus
c9_CT
c9_HT
c9_CM
c9_HM
c9_CF
c9_HF
Start
c9.1
c9.2
c9.3
c9.4
c9.5
c9.6
c9.7
c9.8
c9.9
c9.10
c9.11
c9.12
c9.13
c9.14
c9.15

Figure S3: Genetic and physical maps of chromosomes 2-9. Comparison of the physical and consensus genetic maps with all the different populations: Total meiosis control (CT), total meiosis heat shock (HT), control male meiosis (CM), heat treated male meiosis (HM), control female meiosis (CF), and heat treated female meiosis (HF). The approximate position of the centromere is marked with a red bar. The physical chromosome map shows the proportional position of the markers when physical chromosome is scaled to the same size as the corresponding consensus map (units not in Mbp).

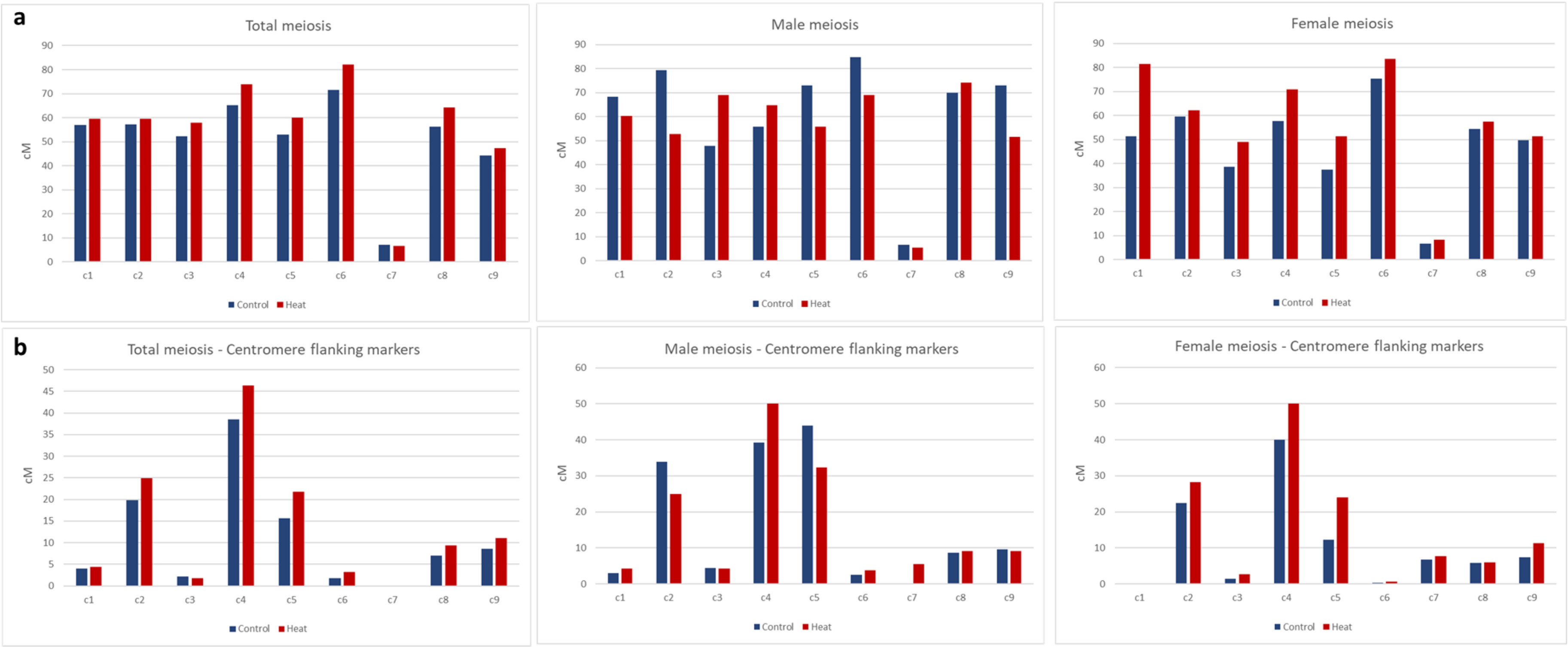

Figure S4: Total recombination per chromosome and population (a), and recombination between centromere flanking markers per chromosome and population (b).

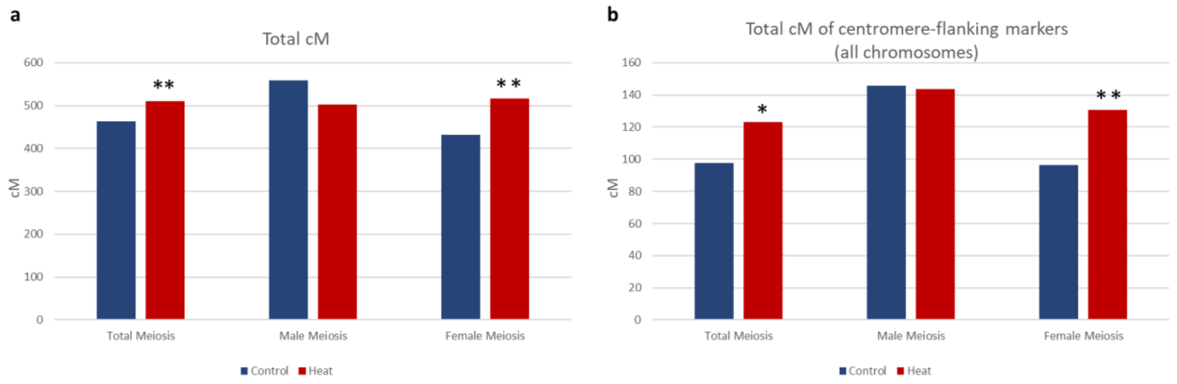

Figure S5: Total map distances per population and treatment (a). Recombination between centromere-flanking markers per population and treatment (b). Differences between maps were compared to the control in each case, using the Wilcoxon signed rank test, being * P<0.05 and ** P<0.01.

```
> compare_means(gperseeds ~ Treatment, group.by =  "Meiosis",method = "t.test", data = mydata)
# A tibble: 3 x 9
  Meiosis        .y.       group1  group2       p  p.adj p.format p.signif method
  <fct>          <chr>     <chr>   <chr>    <dbl>  <dbl> <chr>    <chr>    <chr>
1 Total Meiosis  gperseeds Control Heat   0.00248 0.0074 0.0025   **       T-test
2 Male Meiosis   gperseeds Control Heat   0.631   1      0.6305   ns       T-test
3 Female Meiosis gperseeds Control Heat   0.676   1      0.6755   ns       T-test
>
```

Table S1: t-test for comparing see weight (g) between treatments and meiosis.

		Total Meiosis														Male Meiosis												Female Meiosis											
		Control							Heat							Control						Heat						Control						Heat					
Chr.	SNP	A	B	H	NA	χ2	p-value		A	B	H	NA	χ2	p-value		B	H	NA	χ2	p-value		B	H	NA	χ2	p-value		B	H	NA	χ2	p-value		B	H	NA	χ2	p-value	
c1	1	125	128	263	4	0.2	0.89		109	140	260	11	4.0	0.13		153	280	4	37.2	0.00	*****	95	116	2	2.1	0.15		173	180	1	0.1	0.71		87	67	2	2.6	0.11	
c1	2	129	126	261	4	0.1	0.95		107	125	277	11	5.3	0.07		208	223	6	0.5	0.47		99	110	4	0.6	0.45		166	188	0	1.4	0.24		83	71	2	0.9	0.33	
c1	3	125	138	253	4	0.8	0.65		122	120	269	9	1.4	0.49		211	221	5	0.2	0.63		107	104	2	0.0	0.84		167	187	0	1.1	0.29		81	73	2	0.4	0.52	
c1	4	117	137	253	13	1.6	0.45		116	124	276	4	2.8	0.25		201	222	14	1.0	0.31		106	99	8	0.2	0.62		159	190	5	2.8	0.10		78	71	7	0.3	0.57	
c1	5	118	138	262	2	1.6	0.45		120	127	269	4	1.1	0.57		206	225	6	0.8	0.36		111	100	2	0.6	0.45		160	192	2	2.9	0.09		77	77	2	0.0	1.00	
c1	6	117	143	257	3	2.6	0.27		119	127	261	13	0.7	0.71		209	224	4	0.5	0.47		109	102	2	0.2	0.63		164	190	0	1.9	0.17		70	84	2	1.3	0.26	
c1	7	118	134	265	3	1.3	0.52		122	122	267	9	1.0	0.60		211	219	7	0.1	0.70		107	103	3	0.1	0.78		165	189	0	1.6	0.20		61	91	4	5.9	0.01	*
c1	8	114	134	267	5	2.3	0.32		119	117	274	10	2.8	0.24		208	220	9	0.3	0.56		102	107	4	0.1	0.73		160	183	11	1.5	0.21		61	76	19	1.6	0.20	
c1	9	113	132	270	5	2.6	0.27		125	116	261	18	1.1	0.57		216	216	5	0.0	1.00		109	102	2	0.2	0.63		162	191	1	2.4	0.12		68	86	2	2.1	0.15	
c1	10	114	131	273	2	2.6	0.27		127	120	269	4	1.1	0.57		215	217	5	0.0	0.92		108	102	3	0.2	0.68		162	192	0	2.5	0.11		67	87	2	2.6	0.11	
c1	11	108	134	263	15	3.6	0.17		129	111	250	30	1.5	0.47		221	210	6	0.3	0.60		108	103	2	0.1	0.73		165	186	3	1.3	0.26		67	85	4	2.1	0.14	
c1	12	104	134	278	4	6.6	0.04	*	136	123	257	4	0.7	0.72		219	214	4	0.1	0.81		107	104	2	0.0	0.84		163	191	0	2.2	0.14		54	100	2	13.7	0.00	***
c2	76	136	124	259	1	0.6	0.76		124	124	264	8	0.5	0.78		208	224	5	0.6	0.44		105	105	3	0.0	1.00		175	179	0	0.0	0.83		78	76	2	0.0	0.87	
c2	77	137	119	246	18	1.5	0.47		118	124	267	11	1.4	0.50		136	290	11	55.7	0.00	*****	109	100	4	0.4	0.53		158	178	18	1.2	0.28		77	74	5	0.1	0.81	
c2	78	137	115	252	16	1.9	0.38		129	121	262	8	0.5	0.77		138	291	8	54.6	0.00	*****	111	98	4	0.8	0.37		167	175	12	0.2	0.67		81	70	5	0.8	0.37	
c2	79	127	141	240	12	2.3	0.31		155	109	246	10	8.9	0.01	*	224	203	10	1.0	0.31		107	98	8	0.4	0.53		178	169	7	0.2	0.63		79	75	2	0.1	0.75	
c2	80	123	138	235	24	2.3	0.32		156	103	248	13	11.3	0.00	**	221	196	20	1.5	0.22		109	99	5	0.5	0.49		172	168	14	0.0	0.83		78	73	5	0.2	0.68	
c2	81	128	144	247	1	2.2	0.33		149	110	257	4	5.9	0.05		218	214	5	0.0	0.85		107	104	2	0.0	0.84		189	165	0	1.6	0.20		77	77	2	0.0	1.00	
c2	82	121	142	256	1	1.8	0.41		133	112	269	6	2.8	0.24		219	213	5	0.1	0.77		116	93	4	2.5	0.11		189	165	0	1.6	0.20		73	80	3	0.3	0.57	
c2	83	121	142	256	1	1.8	0.41		134	113	272	1	2.9	0.23		221	209	7	0.3	0.56		114	94	5	1.9	0.17		187	165	2	1.4	0.24		73	81	2	0.4	0.52	
c2	84	124	136	252	8	0.7	0.71		130	111	262	17	2.3	0.31		212	218	7	0.1	0.77		115	94	4	2.1	0.15		195	158	1	3.9	0.05	*	73	80	3	0.3	0.57	
c2	85	122	136	256	6	0.8	0.68		132	117	261	10	1.2	0.56		214	212	11	0.0	0.92		108	101	4	0.2	0.63		194	156	4	4.1	0.04	*	70	81	5	0.8	0.37	
c2	86	123	139	257	1	1.0	0.60		133	119	260	8	0.9	0.64		218	215	4	0.0	0.89		110	101	2	0.4	0.54		197	157	0	4.5	0.03	*	71	83	2	0.9	0.33	
c3	31	132	128	253	7	0.2	0.92		123	125	260	12	0.3	0.86		227	206	4	1.0	0.31		53	158	2	52.3	0.00	*****	186	160	8	2.0	0.16		83	68	5	1.5	0.22	
c3	32	134	127	255	4	0.3	0.88		120	121	269	10	1.5	0.46		226	208	3	0.7	0.39		94	115	4	2.1	0.15		182	149	23	3.3	0.07		83	69	4	1.3	0.26	
c3	33	140	128	244	8	1.7	0.43		119	119	278	4	3.1	0.21		232	194	11	3.4	0.07		98	112	3	0.9	0.33		177	156	21	1.3	0.25		84	68	4	1.7	0.19	
c3	34	138	122	260	0	1.0	0.61		133	114	268	5	2.3	0.32		250	184	3	10.0	0.00	**	101	110	2	0.4	0.54		184	168	2	0.7	0.39		78	76	2	0.0	0.87	
c3	35	141	121	255	3	1.6	0.44		123	112	277	8	3.9	0.14		254	176	7	14.1	0.00	***	102	110	1	0.3	0.58		171	165	18	0.1	0.74		79	75	2	0.1	0.75	
c3	36	133	103	278	6	6.9	0.03	*	133	116	265	6	1.6	0.44		257	173	7	16.4	0.00	****	100	109	4	0.4	0.53		177	175	2	0.0	0.92		78	74	4	0.1	0.75	
c3	37	119	110	291	0	7.7	0.02	*	143	129	247	1	2.0	0.38		262	172	3	18.7	0.00	****	98	112	3	0.9	0.33		177	177	0	0.0	1.00		80	74	2	0.2	0.63	
c3	38	118	112	290	0	7.1	0.03	*	145	129	245	1	2.6	0.27		264	168	5	21.3	0.00	*****	99	111	3	0.7	0.41		176	178	0	0.0	0.92		81	73	2	0.4	0.52	
c4	47	111	130	261	18	2.2	0.33		132	125	261	2	0.2	0.90		220	196	21	1.4	0.24		106	103	4	0.0	0.84		167	175	12	0.2	0.67		63	88	5	4.1	0.04	*
c4	48	114	117	282	7	5.1	0.08		172	115	229	4	19.1	0.00	****	219	213	5	0.1	0.77		108	102	3	0.2	0.68		186	163	5	1.5	0.22		104	49	3	19.8	0.00	*****
c4	49	113	118	284	5	5.6	0.06		169	116	229	6	17.0	0.00	***	220	213	4	0.1	0.74		107	103	3	0.1	0.78		188	164	2	1.6	0.20		103	50	3	18.4	0.00	****
c4	50	106	116	285	13	8.2	0.02	*	167	114	229	10	16.3	0.00	***	206	212	19	0.1	0.77		104	102	7	0.0	0.89		177	161	16	0.8	0.38		103	45	8	22.7	0.00	*****
c4	51	113	114	286	7	6.8	0.03	*	167	120	227	6	15.6	0.00	***	216	214	7	0.0	0.92		106	105	2	0.0	0.95		172	170	12	0.0	0.91		104	48	4	20.6	0.00	*****
c4	52	114	124	279	3	3.6	0.16		156	125	236	3	7.6	0.02	*	216	217	4	0.0	0.96		107	104	2	0.0	0.84		181	173	0	0.2	0.67		108	45	3	25.9	0.00	*****
c4	53	121	129	263	7	0.6	0.75		141	109	259	11	4.2	0.12		219	214	4	0.1	0.81		113	97	3	1.2	0.27		189	165	0	1.6	0.20		105	49	2	20.4	0.00	*****
c5	69	147	122	238	13	4.4	0.11		143	122	249	6	2.2	0.33		200	217	20	0.7	0.41		84	121	8	6.7	0.01	**	181	152	21	2.5	0.11		60	92	4	6.7	0.01	**
c5	70	128	104	250	38	3.1	0.22		107	93	256	64	7.7	0.02	*	206	227	4	1.0	0.31		87	122	4	5.9	0.02	*	193	161	0	2.9	0.09		60	93	3	7.1	0.01	**
c5	71	146	119	236	19	4.6	0.10		142	126	246	6	1.9	0.38		198	226	13	1.8	0.17		83	123	7	7.8	0.01	**	185	154	15	2.8	0.09		57	96	3	9.9	0.00	**
c5	72	148	123	242	7	4.1	0.13		143	124	243	10	2.5	0.28		201	224	12	1.2	0.26		85	125	3	7.6	0.01	**	188	158	8	2.6	0.11		58	95	3	8.9	0.00	**
c5	73	156	109	216	39	14.2	0.00	***	126	112	203	79	3.7	0.16		208	219	10	0.3	0.59		87	121	5	5.6	0.02	*	192	153	9	4.4	0.04	*	62	90	4	5.2	0.02	*
c5	74	186	70	256	8	52.6	0.05	*	147	113	248	12	4.8	0.09		112	318	7	98.7	0.00	*****	48	163	2	62.7	0.00	*****	184	167	3	0.8	0.36		53	98	5	13.4	0.00	***
c5	75	182	81	255	2	39.5	0.00	*****	151	111	255	3	6.3	0.04	*	110	322	5	104.0	0.00	*****	50	161	2	58.4	0.00	*****	176	177	1	0.0	0.96		47	107	2	23.4	0.00	*****
c6	33	138	121	255	6	1.2	0.56		124	128	263	5	0.3	0.86		212	219	6	0.1	0.74		103	108	2	0.1	0.73		196	157	1	4.3	0.04	*	54	100	2	13.7	0.00	***
c6	34	139	122	257	2	1.1	0.56		122	128	261	9	0.4	0.83		214	220	3	0.1	0.77		103	107	3	0.1	0.78		193	156	5	3.9	0.05	*	54	99	3	13.2	0.00	***
c6	35	135	117	262	6	1.5	0.48		144	131	240	5	3.0	0.22		211	219	7	0.1	0.70		109	102	2	0.2	0.63		204	148	2	8.9	0.00	**	74	78	4	0.1	0.75	
c6	36	138	130	245	7	1.3	0.53		136	126	242	16	1.2	0.55		213	219	5	0.1	0.77		101	109	3	0.3	0.58		191	146	17	6.0	0.01	*	80	75	1	0.2	0.69	
c6	37	132	138	247	3	1.2	0.56		137	134	242	7	1.7	0.43		207	224	6	0.7	0.41		111	101	1	0.5	0.49		198	154	2	5.5	0.02	*	82	72	2	0.6	0.42	
c6	38	130	136	252	2	0.5	0.77		145	134	240	1	3.4	0.18		202	231	4	1.9	0.16		109	100	4	0.4	0.53		196	158	0	4.1	0.04	*	79	73	4	0.2	0.63	
c6	39	124	133	255	8	0.3	0.85		142	134	241	3	2.6	0.27		203	228	6	1.5	0.23		107	99	7	0.3	0.58		197	154	3	5.3	0.02	*	70	83	3	1.1	0.29	
c6	40	126	128	265	1	0.2	0.88		137	134	243	6	1.6	0.46		201	232	4	2.2	0.14		109	101	3	0.3	0.58		194	160	0	3.3	0.07		70	84	2	1.3	0.26	
c6	41	132	120	266	2	0.9	0.63		146	116	252	6	3.7	0.16		207	222	8	0.5	0.47		121	90	2	4.6	0.03	*	192	160	2	2.9	0.09		73	81	2	0.4	0.52	
c6	42	131	119	266	4	1.1	0.59		146	116	252	6	3.7	0.16		208	224	5	0.6	0.44		121	87	5	5.6	0.02	*	193	159	2	3.3	0.07		75	80	1	0.2	0.69	
c6	43	133	120	264	3	0.9	0.64		146	115	251	8	3.9	0.14		209	225	3	0.6	0.44		121	91	1	4.2	0.04	*	189	160	5	2.4	0.12		73	80	3	0.3	0.57	
c6	44	128	123	259	10	0.2	0.89		149	115	252	4	4.8	0.09		211	219	7	0.1	0.70		116	90	7	3.3	0.07		190	161	3	2.4	0.12		72	82	2	0.6	0.42	
c6	45	125	128	260	7	0.1	0.94		150	120	244	6	4.8	0.09		210	223	4	0.4	0.53		116	93	4	2.5	0.11		191	159	4	2.9	0.09		70	84	2	1.3	0.26	
c6	46	118	135	266	1	1.4	0.49		127	120	270	3	1.2	0.55		114	320	3	97.8	0.00	*****	103	106	4	0.0	0.84		183	170	1	0.5	0.49		61	91	4	5.9	0.01	*
c7	28	124	134	257	5	0.4	0.82		129	137	251	3	0.7	0.71		169	263	5	20.5	0.00	*****	51	158	4	54.8	0.00	*****	167	184	3	0.8	0.36		59	92	5	7.2	0.01	**
c7	29	124	132	257	7	0.3	0.88		128	133	248	11	0.4	0.81		170	262	5	19.6	0.00	*****	51	159	3	55.5	0.00	*****	165	184	5	1.0	0.31		63	92	1	5.4	0.02	*
c7	30	113	127	271	9	2.6	0.27		126	127	257	10	0.0	0.98		178	238	21	8.7	0.00	**	51	152	10	50.3	0.00	*****	163	171	20	0.2	0.66		59	87	10	5.4	0.02	*
c8	54	115	147	255	3	4.1	0.13		121	136	262	1	0.9	0.63		206	224	7	0.8	0.39		106	103	4	0.0	0.84		178	176	0	0.0	0.92		80	74	2	0.2	0.63	
c8	55	110	144	258	8	4.5	0.10		121	135	261	3	0.8	0.67		206	219	12	0.4	0.53		105	107	1	0.0	0.89		175	169	10	0.1	0.75		79	73	4	0.2	0.63	
c8	56	113	137	267	3	2.8	0.25		107	140	270	3	5.2	0.07		199	231	7	2.4	0.12		105	103	5	0.0	0.89		182	171	1	0.3	0.56		78	76	2	0.0	0.87	
c8	57	104	140	274	2	6.7	0.03	*	103	136	272	9	6.4	0.04	*	200	232	5	2.4	0.12		104	107	2	0.0	0.84		182	166	6	0.7	0.39		75	78	3	0.1	0.81	
c8	58	104	145	265	6	7.0	0.03	*	110	135	271	4	3.7	0.15		199	233	5	2.7	0.10		102	107	4	0.1	0.73		181	170	3	0.3	0.56		74	80	2	0.2	0.63	
c8	59	110	146	258	6	5.1	0.08		111	141	260	8	3.6	0.16		200	231	6	2.2	0.14		104	104	5	0.0	1.00		189	165	0	1.6	0.20		71	82	3	0.8	0.37	
c8	60	113	146	259	2	4.2	0.12		131	141	247	1	1.6	0.45		198	236	3	3.3	0.07		101	110	2	0.4	0.54		191	162	1	2.4	0.12		75	79	2	0.1	0.75	
c8	61	108	141	269	2	5.0	0.08		134	134	250	2	0.6	0.73		188	239	10	6.1	0.01	*	96	115	2	1.7	0.19		186	160	8	2.0	0.16		74	79	3	0.2	0.69	
c8	62	109	135	272	4	4.1	0.13		128	119	259	14	0.6	0.74		192	240	5	5.3	0.02	*	89	123	1	5.5	0.02	*	184	166	4	0.9	0.34		72	84	0	0.9	0.34	
c8	63	107	133	272	8	4.6	0.10		132	123	262	3	0.4	0.82		189	237	11	5.4	0.02	*	88	116	9	3.8	0.05	*	182	163	9	1.0	0.31		69	82	5	1.1	0.29	
c8	64	106	132	276	6	5.4	0.07		134	121	260	5	0.7	0.70		194	238	5	4.5	0.03	*	89	120	4	4.6	0.03	*	187	167	0	1.1	0.29		71	83	2	0.9	0.33	
c8	65	107	131	280	2	5.6	0.06		135	124	258	3	0.5	0.79		199	237	1	3.3	0.07		92	119	2	3.5	0.06		188	166	0	1.4	0.24		70	84	2	1.3	0.26	
c8	66	104	128	279	9	6.6	0.04	*	133	124	261	2	0.3	0.84		197	229	11	2.4	0.12		89	115	9	3.3	0.07		179	158	17	1.3	0.25		69	82	5	1.1	0.29	
c8	67	120	139	259	2	1.4	0.50		132	120	261	7	0.7	0.70		148	282	7	41.8	0.00	*****	47	164	2	64.9	0.00	*****	129	130	95	0.0	0.95		44	62	2	3.1	0.08	
c8	68	118	137	258	7	1.4	0.49		132	120	264	4	0.8	0.66		149	278	10	39.0	0.00	*****	48	164	1	63.5	0.00	*****	127	128	99	0.0	0.95		44	63	1	3.4	0.07	
c9	13	125	117	275	3	2.4	0.31		133	130	249	8	0.4	0.81		261	172	4	18.3	0.00	****	102	109	2	0.2	0.63		191	161	2	2.6	0.11		78	76	2	0.0	0.87	
c9	14	127	115	270	8	2.1	0.35		132	127	240	21	0.8	0.66		260	172	5	17.9	0.00	****	102	108	3	0.2	0.68		188	161	5	2.1	0.15		79	76	1	0.1	0.81	
c9	15	127	119	273	1	1.7	0.44		135	130	251	4	0.5	0.79		260	172	5	17.9	0.00	****	100	109	4	0.4	0.53		191	161	2	2.6	0.11		78	76	2	0.0	0.87	
c9	16	126	118	271	5	1.7	0.44		134	131	252	3	0.4	0.83		259	172	6	17.6	0.00	****	101	109	3	0.3	0.58		190	160	4	2.6	0.11		78	76	2	0.0	0.87	
c9	17	126	117	274	3	2.2	0.34		134	131	252	3	0.4	0.83		261	170	6	19.2	0.00	****	102	108	3	0.2	0.68		190	162	2	2.2	0.14		78	76	2	0.0	0.87	
c9	18	124	117	272	7	2.1	0.36		132	130	252	6	0.2	0.90		261	172	4	18.3	0.00	****	101	108	4	0.2	0.63		191	161	2	2.6	0.11		78	76	2	0.0	0.87	
c9	19	123	115	269	13	2.1	0.34		131	125	244	20	0.4	0.81		258	173	6	16.8	0.00	****	102	109	2	0.2	0.63		189	158	7	2.8	0.10		78	76	2	0.0	0.87	
c9	20	129	123	267	1	0.6	0.75		131	131	255	3	0.1	0.95		260	172	5	17.9	0.00	****	96	113	4	1.4	0.24		192	162	0	2.5	0.11		81	73	2	0.4	0.52	
c9	21	124	120	250	26	0.1	0.93		125	123	234	38	0.4	0.81		259	177	1	15.4	0.00	****	97	113	3	1.2	0.27		188	159	7	2.4	0.12		83	71	2	0.9	0.33	
c9	22	134	117	267	2	1.6	0.45		140	129	248	3	1.3	0.52		188	244	5	7.3	0.01	**	98	109	6	0.6	0.44		190	161	3	2.4	0.12		81	73	2	0.4	0.52	
c9	23	138	111	270	1	3.7	0.16		144	112	259	5	4.0	0.14		186	244	7	7.8	0.01	**	103	107	3	0.1	0.78		186	167	1	1.0	0.31		76	78	2	0.0	0.87	
c9	24	135	111	270	4	3.3	0.19		138	114	252	16	2.3	0.32		181	252	4	11.6	0.00	***	102	108	3	0.2	0.68		181	173	0	0.2	0.67		75	77	4	0.0	0.87	
c9	25	136	105	271	8	5.5	0.06		142	111	250	17	3.8	0.15		247	187	3	8.3	0.00	**	106	106	1	0.0	1.00		183	169	2	0.6	0.46		76	78	2	0.0	0.87	
c9	26	138	105	274	3	6.1	0.05	*	143	102	271	4	7.8	0.02	*	231	200	6	2.2	0.14		104	104	5	0.0	1.00		171	179	4	0.2	0.67		79	73	4	0.2	0.63	
c9	27	137	101	276	6	7.9	0.02	*	140	106	260	14	5.0	0.08		235	197	5	3.3	0.07		105	106	2	0.0	0.95		169	181	4	0.4	0.52		83	69	4	1.3	0.26	

Table S2: Segregation of alleles across the different loci. Intervals in yellow show markers that flank the centromere. The significant distortion is highlighted in red *, P ≤ 0.05; **, P ≤ 0.01;***, P ≤ 0.001; ****, P ≤ 0.0001; *****, P ≤ 0.00001).

Tests for fixed effects

Sequentially adding terms to fixed model

Fixed term	Wald statistic	n.d.f.	F statistic	d.d.f.	F pr
treatment	11.53	1	11.53	70.1	0.001
chromosome	12.57	7	1.80	67.6	0.102
chromosome.treatment	4.18	7	0.60	70.1	0.756

Dropping individual terms from full fixed model

Fixed term	Wald statistic	n.d.f.	F statistic	d.d.f.	F pr
chromosome.treatment	4.18	7	0.60	70.1	0.756

Table S3: Output of the GLMM in total meiosis to test the effect on recombination of treatment and chromosome as fixed effects, and marker interval as random effect among all chromosomes together, and among the intervals within each chromosome. The significant term is highlighted in yellow.

Tests for fixed effects

Sequentially adding terms to fixed model

Fixed term	Wald statistic	n.d.f.	F statistic	d.d.f.	F pr
sex	4.72	1	4.72	222.8	0.031
treatment	0.33	1	0.33	222.8	0.565
chromosome	7.50	7	1.07	66.6	0.392
sex.treatment	6.41	1	6.41	222.8	0.012
chromosome.sex	8.17	7	1.17	222.8	0.323
chromosome.treatment	8.29	7	1.18	222.8	0.313
chromosome.sex.treatment	4.88	7	0.70	222.8	0.675

Dropping individual terms from full fixed model

Fixed term	Wald statistic	n.d.f.	F statistic	d.d.f.	F pr
chromosome.sex.treatment	4.88	7	0.70	222.8	0.675

Table S4: Output of the GLMM for male and female meiosis to test the effect on recombination of treatment , sex, and chromosome as fixed effects, and marker interval as random effect among all chromosomes together, and among the intervals within each chromosome. The significant term is highlighted in yellow.

```
> compare_means(cM ~ Treat, group.by = c("Population","Recombination"), data = mydata, paired = TRUE)
# A tibble: 6 x 10
  Population     Recombination              .y.   group1  group2        p p.adj p.format p.signif method  
  <chr>          <chr>                      <chr> <chr>   <chr>     <dbl> <dbl> <chr>    <chr>    <chr>   
1 Total Meiosis  Total cM per chromosome    cM    Control Heat    0.00781 0.039 0.0078   **       Wilcoxon
2 Male Meiosis   Total cM per chromosome    cM    Control Heat    0.301   0.6   0.3008   ns       Wilcoxon
3 Female Meiosis Total cM per chromosome    cM    Control Heat    0.00391 0.023 0.0039   **       Wilcoxon
4 Total Meiosis  Centromere flanking markers cM   Control Heat    0.0209  0.063 0.0209   *        Wilcoxon
5 Male Meiosis   Centromere flanking markers cM   Control Heat    0.734   0.73  0.7344   ns       Wilcoxon
6 Female Meiosis Centromere flanking markers cM   Control Heat    0.0143  0.057 0.0143   *        Wilcoxon
```

Table S5: Wilcoxon signed rank test for comparing recombination between treatments.

Marker Name	flanking centromere	Total Meiosis				Male meiosis				Female Meiosis			
		Control		Heat Stress		Control		Heat Stress		Control		Heat Stress	
		cM	cM Map	cM	cM Map	cM	cM Map	cM	cM Map	cM	cM Map	cM	cM Map
c1.1	left	8.96	0.00	7.8	0.00	20.58	0.00	5.28	0.00	10.34	0.00	7.86	0.00
c1.2	left	11.47	8.96	11.3	7.81	8.93	20.58	13.73	5.28	11.80	10.34	6.53	7.86
c1.3	left	3.34	20.43	3.3	19.06	4.99	29.51	3.42	19.02	3.44	22.14	3.36	14.39
c1.4	left	3.84	23.77	3.5	22.38	6.69	34.50	5.88	22.43	3.75	25.58	6.07	17.75
c1.5	left	4.78	27.61	4.0	25.85	4.65	41.19	7.64	28.32	3.41	29.34	7.19	23.82
c1.6	left	6.79	32.39	8.1	29.82	6.55	45.85	6.71	35.96	5.39	32.75	11.38	31.01
c1.7	left	1.58	39.18	1.9	37.96	1.41	52.39	1.91	42.66	0.58	38.14	3.68	42.39
c1.8	left	5.88	40.76	6.3	39.88	4.71	53.81	5.28	44.58	4.38	38.72	12.67	46.07
c1.9	left	0.10	46.64	0.0	46.13	0.23	58.52	0.00	49.86	3.43	43.11	3.96	58.74
c1.10	left ->	3.97	46.73	4.5	46.13	3.04	58.75	4.30	49.86	0.00	46.54	0.00	62.70
c1.11	right <-	6.22	50.70	8.9	50.60	6.56	61.79	6.19	54.16	4.86	46.54	18.71	62.70
c1.12	right	0.0	56.92	0.0	59.51	0.0	68.35	0.0	60.35	0.0	51.40	0.0	81.41
		4.0	56.9	4.5	59.5	3.0	68.4	4.3	60.4	0.0	51.4	0.0	81.4
c2.1	left	3.7	0.00	3.1	0.00	35.7	0.00	4.9	0.00	4.8	0.00	3.3	0.00
c2.2	left	3.5	3.67	3.4	3.14	19.4	35.70	2.9	4.87	3.0	4.78	2.7	3.32
c2.3	left	31.3	7.17	30.3	6.57	3.3	55.05	26.6	7.80	35.5	7.81	36.8	6.02
c2.4	left	1.1	38.44	1.0	36.85	0.7	58.39	0.5	34.44	1.2	43.36	1.3	42.82
c2.5	left	4.0	39.57	4.9	37.86	5.6	59.13	4.4	34.94	1.5	44.55	0.7	44.15
c2.6	left ->	8.6	43.60	11.1	42.76	9.6	64.70	9.2	39.30	7.4	46.02	11.4	44.82
c2.7	right <-	0.4	52.24	0.1	53.84	0.2	74.33	0.0	48.49	0.0	53.41	0.0	56.19
c2.8	right	2.6	52.62	2.8	53.94	1.6	74.57	4.3	48.49	3.1	53.41	1.3	56.19
c2.9	right	1.9	55.20	2.8	56.77	3.3	76.21	0.0	52.83	3.1	56.55	4.7	57.50
c2.10	right	0.0	57.10	0.0	59.55	0.0	79.47	0.0	52.83	0.0	59.67	0.0	62.18
c2.11	right	0.0	57.10	0.0	59.55	0.0	79.47	0.0	52.83	0.0	59.67	0.0	62.18
		8.6	57.1	11.1	59.6	9.6	79.5	9.2	52.8	7.4	59.7	11.4	62.2
c3.1	left	7.1	0.00	5.4	0.00	6.3	0.00	25.6	0.00	6.4	0.00	6.8	0.00
c3.2	left	5.0	7.14	5.5	5.38	5.4	6.28	3.4	25.58	4.4	6.42	7.4	6.80
c3.3	left	12.0	12.10	12.8	10.87	11.7	11.72	17.4	28.96	12.0	10.80	10.1	14.24
c3.4	left	4.8	24.15	7.1	23.72	6.1	23.46	6.7	46.38	8.2	22.81	9.3	24.30
c3.5	left	4.6	28.92	6.1	30.82	12.7	29.55	10.2	53.09	5.4	30.97	12.2	33.56
c3.6	left	1.5	33.51	2.4	36.88		42.29		63.33		36.38		45.71
c3.7	left	0.0	35.04	0.4	39.27		42.29		63.33		36.38		45.71
c3.8	left	0.2	35.04	0.5	39.69		42.29		63.33		36.38		45.71
c3.9	left	0.0	35.24	0.0	40.22		42.29		63.33		36.38		45.71
c3.10	left	1.0	35.24	0.6	40.22		42.29		63.33		36.38		45.71
c3.11	left	0.1	36.22	0.0	40.81		42.29		63.33		36.38		45.71
c3.12	left ->	1.5	36.31	1.0	40.81		42.29		63.33		36.38		45.71
c3.13	cent	0.7	37.79	0.8	41.79	4.4	42.29	4.3	63.33	1.4	36.38	2.6	45.71
c3.14	right <-	4.2	38.48	4.7	42.60		46.72		67.66		37.80		48.35
c3.15	right	1.1	42.67	1.2	47.29	1.2	46.72	1.4	67.66	0.8	37.80	0.0	48.35
c3.16	right	8.6	43.73	9.5	48.46	0.0	47.87	0.0	69.09	0.0	38.65	0.6	48.35
c3.17	right	0.0	52.35	0.0	57.99		47.87		69.09		38.65		49.00
		2.2	52.3	1.8	58.0	4.4	47.9	4.3	69.1	1.4	38.6	2.6	49.0

c4.1	left	1.6	0.00	1.0	0.00		0.00		0.00		0.00		0.00
c4.2	left	0.1	1.58	0.1	0.98		0.00		0.00		0.00		0.00
c4.3	left	0.0	1.68	0.0	1.08		0.00		0.00		0.00		0.00
c4.4	left	7.3	1.68	7.3	1.08		0.00		0.00		0.00		0.00
c4.5	left	38.5	8.94	46.4	8.41	39.2	0.00	50.0	0.00	40.1	0.00	50.0	0.00
c4.6	right <-	0.6	47.48	0.4	54.80	0.9	39.23	0.0	50.00	2.1	40.06	2.0	50.00
c4.7	right	2.6	48.07	1.2	55.20	1.4	40.16	7.2	50.00	0.0	42.15	0.0	52.04
c4.8	right	3.5	50.70	5.4	56.39	5.1	41.60	0.0	57.23	3.0	42.15	3.5	52.04
c4.9	right	2.5	54.16	3.4	61.75	2.8	46.71	1.0	57.23	4.1	45.18	2.7	55.50
c4.10	right	8.6	56.64	8.7	65.14	6.3	49.51	6.7	58.18	8.6	49.28	12.7	58.16
c4.11	right	0.0	65.25	0.0	73.88	0.0	55.80	0.0	64.89	0.0	57.84	0.0	70.85
		38.5	65.2	46.4	73.9	39.2	55.8	50.0	64.9	40.1	57.8	50.0	70.8
c5.1	left	1.6	0.00	3.0	0.00	0.0	0.00	0.0	0.00	0.3	0.00	0.0	0.00
c5.2	left	4.8	1.60	3.5	3.05	4.0	0.00	2.5	0.00	4.4	0.30	5.3	0.00
c5.3	left	1.1	6.39	0.5	6.54	0.5	4.04	1.0	2.45	0.6	4.74	2.7	5.32
c5.4	left	5.4	7.50	5.0	7.03	14.0	4.52	12.4	3.44	9.0	5.34	10.1	7.97
c5.5	left	2.9	12.94	2.9	12.08		18.54		15.88		14.31		18.11
c5.6	left	3.4	15.85	4.0	14.99		18.54		15.88		14.31		18.11
c5.7	left ->	15.7	19.30	21.8	18.99	44.0	18.54	32.4	15.88	12.2	14.31	24.0	18.11
c5.8	right <-	7.1	35.00	8.2	40.74		62.52		48.26		26.50		42.09
c5.9	right	10.8	42.15	11.1	48.93	10.4	62.52	7.6	48.26	11.0	26.50	9.4	42.09
c5.10	right	0.0	52.97	0.0	60.05	0.0	72.92	0.0	55.91	0.0	37.53	0.0	51.47
		15.7	53.0	21.8	60.1	44.0	72.9	32.4	55.9	12.2	37.5	24.0	51.5
c6.1	left	0.4	0.00	0.3	0.00	0.2	0.00	10.6	0.00	0.0	0.00	20.1	0.00
c6.2	left	11.8	0.39	12.1	0.30	10.9	0.23	0.0	10.58	8.4	0.00	0.0	20.10
c6.3	left	8.7	12.23	7.1	12.39	8.7	11.10	7.3	10.58	8.4	8.44	8.0	20.10
c6.4	left	2.8	20.97	2.9	19.46	1.6	19.83	3.8	17.84	2.1	16.87	1.9	28.06
c6.5	left	0.8	23.75	0.9	22.32	1.4	21.46	0.0	21.68	1.4	18.96	0.7	30.01
c6.6	left	2.4	24.53	3.3	23.20	4.0	22.86	4.4	21.68	4.0	20.38	8.0	30.67
c6.7	left	1.9	26.91	2.2	26.55	2.6	26.82	1.5	26.10	3.4	24.38	2.6	38.68
c6.8	left	10.3	28.78	13.0	28.72	17.4	29.38	21.0	27.57	13.5	27.80	14.2	41.30
c6.9	left	4.1	39.11	10.0	41.76		46.79		48.52		41.30		55.48
c6.10	left	1.4	43.20	3.9	51.80		46.79		48.52		41.30		55.48
c6.11	left	3.0	44.61	2.7	55.70		46.79		48.52		41.30		55.48
c6.12	left ->	0.3	47.58	0.5	58.36		46.79		48.52		41.30		55.48
c6.13	cent	0.0	47.87	0.0	58.85	2.6	46.79	0.0	48.52	0.3	41.30	0.6	55.48
c6.14	cent	0.0	47.87	0.1	58.85	0.0	49.38	0.0	48.52	0.0	41.59	0.0	56.13
c6.15	cent	1.8	47.87	3.2	58.95	0.0	49.38	3.8	48.52	0.0	41.59	0.0	56.13
c6.16	right <-	0.8	49.66	1.1	62.14	0.7	49.38	16.8	52.35	1.2	41.59	3.3	56.13
c6.17	right	0.1	50.45	0.2	63.23	34.8	50.09	0.0	69.13	32.6	42.74	24.3	59.41
c6.18	right	11.0	50.55	10.2	63.43		84.89		69.13		75.34		83.66
c6.19	right	10.0	61.56	8.5	73.59		84.89		69.13		75.34		83.66
c6.20	right	0.0	71.59	0.0	82.06	0.0	84.89	0.0	69.13	0.0	75.34	0.0	83.66
		1.8	71.6	3.3	82.1	2.6	84.9	3.8	69.1	0.3	75.3	0.6	83.7
c7.1	right	0.0	0.00	0.0	0.00	0.0	0.00	5.5	0.00	6.7	0.00	7.8	0.00
c7.2	right	7.1	0.00	6.5	0.00	6.8	0.00	0.0	5.49	0.0	6.69	0.7	7.75
c7.3	right	0.0	7.14	0.0	6.52	0.0	6.79	0.0	5.49	0.0	6.69	0.0	8.42
		0.0	7.1	0.0	6.5	0.0	6.8	5.5	5.5	6.7	6.7	7.8	8.4

c8.1	left	2.4	0.00	1.6	0.00	2.6	0.00	0.5	0.00	2.9	0.00	2.7	0.00
c8.2	left	13.9	2.39	12.0	1.56	13.7	2.62	8.3	0.48	11.6	2.91	15.7	2.65
c8.3	left	3.4	16.25	3.7	13.59	2.3	16.28	1.0	8.77	6.7	14.48	3.3	18.38
c8.4	left	5.0	19.61	7.4	17.32	3.7	18.61	5.3	9.73	5.2	21.15	5.9	21.66
c8.5	left	4.3	24.65	4.7	24.73	4.7	22.33	2.9	15.01	4.0	26.39	3.9	27.56
c8.6	left ->	5.6	28.98	6.9	29.48	6.8	27.01	6.8	17.93	6.8	30.38	5.9	31.49
c8.7	-	5.3	34.62	6.6	36.39	5.2	33.78	9.1	24.70	4.7	37.23	2.0	37.40
c8.8	-	3.9	39.92	3.6	43.01	8.0	38.96	7.7	33.80	4.6	41.88	3.4	39.37
c8.9	-	1.9	43.79	4.0	46.62		46.92		41.48		46.51		42.73
c8.10	-	0.5	45.72	0.6	50.66		46.92		41.48		46.51		42.73
c8.11	-	0.0	46.21	0.0	51.26	0.7	46.92	0.0	41.48	0.0	46.51	1.9	42.73
c8.12	-	0.7	46.21	1.2	51.26	0.0	47.62	1.5	41.48	1.2	46.51	0.7	44.68
c8.13	right <-	0.6	46.90	0.8	52.44	1.2	47.62	0.5	42.98	0.0	47.73	0.7	45.34
c8.14	right	0.2	47.49	0.4	53.22	0.7	48.78	1.0	43.45	6.6	47.73	11.5	46.00
c8.15	right	1.8	47.69	2.5	53.61	20.5	49.48	29.6	44.44	0.0	54.33	0.0	57.53
c8.16	right	6.9	49.48	8.2	56.07		69.98		74.09		54.33		57.53
c8.17	right	0.0	56.33	0.0	64.26	0.0	69.98	0.0	74.09	0.0	54.33	0.0	57.53
c8.18	right	0.0	56.33	0.0	64.26	0.0	69.98	0.0	74.09	0.0	54.33	0.0	57.53
		7.0	56.3	9.4	64.3	8.7	70.0	9.2	74.1	5.8	54.3	6.0	57.5
c9.1	right	0.0	0.00	0.0	0.00	1.2	0.00	3.8	0.00	0.3	0.00	1.9	0.00
c9.2	right	0.1	0.00	0.1	0.00	0.0	1.16	0.0	3.84	0.0	0.29	0.0	1.95
c9.3	right	0.0	0.10	0.0	0.10	0.0	1.16	0.5	3.84	0.9	0.29	0.0	1.95
c9.4	right	0.1	0.10	0.0	0.10	0.0	1.16	0.0	4.32	0.0	1.14	0.0	1.95
c9.5	right	0.1	0.20	0.0	0.10	0.0	1.16	0.0	4.32	0.0	1.14	0.0	1.95
c9.6	right	0.0	0.29	0.1	0.10	0.0	1.16	0.0	4.32	0.0	1.14	0.0	1.95
c9.7	right	1.9	0.29	2.0	0.20	0.0	1.16	0.0	4.32	0.0	1.14	0.0	1.95
c9.8	right	0.2	2.15	0.1	2.17	0.2	1.16	0.5	4.32	0.0	1.14	1.3	1.95
c9.9	right	6.4	2.35	6.3	2.27	23.7	1.39	6.9	4.80	5.5	1.14	6.6	3.26
c9.10	right <-	15.0	8.80	18.2	8.53	14.4	25.05	18.8	11.71	15.6	6.69	22.9	9.83
c9.11	-	3.5	23.76	4.9	26.72	2.6	39.42	5.3	30.51	4.8	22.27	3.3	32.74
c9.12	-	1.4	27.23	1.7	31.67	17.0	41.99	1.0	35.79	2.0	27.10	2.0	36.05
c9.13	left ->	12.1	28.61	11.8	33.39	12.3	59.01	13.8	36.75	16.4	29.09	10.8	38.03
c9.14	left	3.5	40.68	2.1	45.21	1.6	71.32	1.0	50.55	4.3	45.46	2.7	48.79
c9.15	left	0.0	44.17	0.0	47.32	0.0	72.95	0.0	51.51	0.0	49.80	0.0	51.47
		19.8	44.2	24.9	47.3	34.0	73.0	25.0	51.5	22.4	49.8	28.2	51.5
TOTAL		97.6	463.8	123.1	511.1	145.5	559.0	143.7	503.3	96.3	431.2	130.6	516.0

Table S6: All calculated cM distances (between markers "cM" and accumulated map "cM map"). The markers bounding the centromere are indicated in column 2 as "left" or "right", and the cM between them is indicated in blue under each of the chromosomes, whilst the total cM of the chromosome is indicated in orange.
